# A principled framework to assess information theoretical fitness of brain functional sub-circuits

**Published:** 2024-06-26

**Authors:** Duy Duong-Tran, Nghi Nguyen, Shizhuo Mu, Jiong Chen, Jingxuan Bao, Frederick Xu, Sumita Garai, Jose Cadena-Pico, Alan David Kaplan, Tianlong Chen, Yize Zhao, Li Shen, Joaquín Goñi

**Affiliations:** 1 Department of Biostatistics, Epidemiology, and Informatics (DBEI), Perelman School of Medicine, University of Pennsylvania, Philadelphia, PA, USA; 2 Department of Mathematics, United States Naval Academy, Annapolis, MD, USA; 3 Gonda Multidisciplinary Brain Research Center, Bar-Ilan University, Ramat Gan, Israel; 4 Machine Learning Group, Lawrence Livermore National Laboratory, Livermore, CA, USA; 5 Computational Engineering Division, Lawrence Livermore National Laboratory, Livermore, CA, USA; 6 Department of Computer Science, The University of North Carolina at Chapel Hill; 7 School of Public Health, Yale University, New Heaven, CT, USA; 8 School of Industrial Engineering, Purdue University, West Lafayette, IN, USA; 9 Purdue Institute for Integrative Neuroscience, Purdue University, West Lafayette, IN, USA; 10 Weldon School of Biomedical Engineering, Purdue University, West Lafayette, IN, USA

## Abstract

In systems and network neuroscience, many common practices in brain connectomic analysis are often not properly scrutinized. One such practice is mapping a predetermined set of sub-circuits, like functional networks (FNs), onto subjects’ functional connectomes (FCs) without adequately assessing the information-theoretic appropriateness of the partition. Another practice that goes unchallenged is thresholding weighted FCs to remove spurious connections without justifying the chosen threshold. This paper leverages recent theoretical advances in Stochastic Block Models (SBMs) to formally define and quantify the information-theoretic fitness (e.g., prominence) of a predetermined set of FNs when mapped to individual FCs under different fMRI task conditions. Our framework allows for evaluating any combination of FC granularity, FN partition, and thresholding strategy, thereby optimizing these choices to preserve important topological features of the human brain connectomes. Our results pave the way for the proper use of predetermined FNs and thresholding methods and provide insights for future research in individualized parcellations.

## 1. Introduction

The success of large-scale brain connectomics—which subserves a myriad of neuroimaging research endeavors based on fMRI [30,41,49], MEG [14], and EEG [42]—hinges on choosing representations of functional connectivity that are as well-defined as possible. Functional connectomes (FCs) are often constructed by computing a statistical dependency measure, such as the Pearson correlation coefficient, across all specified pairs of the brain’s regions of interest (ROIs) using the aggregated voxel level blood-oxygen-level-dependent (BOLD) signals. However, constructing FCs from BOLD signals with activation delays (due to inhibitory-excitatory dynamics possibly causing negative ROI correlations) can significantly impact estimates of population-level FCs [41] and the associated functional brain network topological features such as nodes’ centrality [3], global network measures [30], and geometry-topology relation [36]. Recent efforts have focused on improving FC construction by taking into account neuronal signal activation delays [30] and negative correlations [49]. Nonetheless, much effort is still needed to quantify the efficacy of each FC construction framework, especially in terms of preserving the “true” features of the population FCs that shed light on fundamental principles of the brain.

Functional sub-circuits, *e.g.*, functional networks (FNs) [47], and their modularity characteristics [9, 16, 29, 40] are crucial to understanding such fundamental neural principles, including brain complexity [8], differential configurational properties [16], modular structures [29, 39], and information processing [5, 6]. Studies on the modular organizations of the human brain have also informed applied research on aging [10, 28] and disorders including schizophrenia [4]. Moreover, research consistently shows that executive subsystems in the brain are reproducible across many individuals at rest, *e.g.*, [34,47], indicating a widespread application of these FNs in various studies—from control groups [27] to pathological investigations [13] and predicting individual differences [35]. Even so, there have been few (if any) systematic studies addressing the validity of a common and rarely challenged practice in brain connectomics, which is applying one specific set of *a priori* FNs to multiple FCs. In other words, FC processing usually involves mapping an *a priori* fixed set of FNs onto the constructed FCs, across different subjects and fMRI task conditions, without examining whether those mappings are relevant in the information-theoretic sense to the FCs.

Among the many decisions influencing whole-brain functional connectivity estimates like FCs and circuit-level representations like FNs, the choice of brain parcellations, *i.e.*, how nodes in functional brain networks are defined, is undoubtedly one of the most critical steps. [20, 37, 38]. In fact, this choice determines the network topology used in downstream analyses. Recent studies have shown that different levels of parcellation granularity can affect the identification of subject-level FC fingerprints [1, 19]. In an effort to register the raw neuroimaging data into a sequence of increasing granularity, Schaefer and colleagues have recently published a scheme of atlases that increase in network sizes. These parcellations refine the robust set of resting state networks initially identified by Yeo et al. [47], offering various granularity levels for in-depth analysis. Thanks to these advancements, the brain connectivity research community can now explore characteristics of sequential functional brain networks, especially those coupled with the corresponding *a priori* set of FNs.

Regardless of which parcellation scheme is employed during large-scale FC and FN analyses, another common practice in network neuroscience is thresholding (or, more generally, eliminating statistically spurious functional edges) based on some arbitrary rules or research hypotheses. Careful design of the thresholding process is central to ensuring scientific rigor not only in healthy control studies but also in those studying disorders such as schizophrenia [46], unipolar depression, and & bipolar disorder [48]. Unrigorous application of thresholding can therefore undermine the validity of such important studies by affecting downstream analyses, including parametric statistical tests [24] and network characterization [44]. To mitigate such issues, various thresholding strategies have been proposed to retain particular desired attributes of the original weighted networks. These strategies include proportional thresholding aimed at keeping the absolute number of edges across different subjects and tasks [44], modular similarity [48], and percolation aimed at preserving the topological features of the original weighted graph [17]. Spurious edge elimination also involves methods based on wavelets [46], mixture modeling [12], topological data analysis through persistent homology [25, 26], branch-and-bound based algorithms (to study cognitive activity [42]), and orthogonal minimal spanning trees for dynamical functional brain networks [15]. Furthermore, alternatives to thresholding treatment for FCs have also been proposed using hierarchical Bayesian mixture models. [22]. However, this multitude of strategies further complicates the already complex decision-making process of brain data preprocessing and analysis. After all, how can one determine which combination of FC parcellation, FN partitioning, and edge pruning techniques is optimal for their dataset?

This work tackles the complexity posed by that abundance of choices. Our objectives are two-fold: *i)* formalizing and quantify the level of information prominence of a given fixed set of FNs across different subjects and tasks, and *ii)* using the level of prominence as guidance to eliminate spurious functional edges in whole-brain FCs. To do so, we utilize Schaefer parcellations [38] with nine distinct granularity levels, ranging from 100 to 900 nodes in 100-node increments. We first present a formalization of stochastic block models (SBMs) and its relevance to our quest Section 2. We then propose an SBM reconstruction pipeline in Section 3. We wrap up with Results (Section 4) and Discussion (Section 5). Our framework can be generalized to any given pair of an FN partition and a parcellation (*e.g.* [21, 43]).

## 2. A principled framework to assess information theoretical fitness of brain functional sub-circuits/networks

### 2.1. Background

Stochastic Block Models (SBMs) have recently gained traction due to exciting developments in both theoretical and practical domains (see Preliminaries-Stochastic Block Models in Supplementary Information for further details on notations and a brief introduction). Theory-wise, phase transitions in the fundamental limits of community *detection* (or more generally, mesoscopic structures) were discovered through the measure Signal-to-Noise Ratio (SNR) [2]. In the domain of brain connectivity, SBM has demonstrated its advantages in exploring and uncovering diverse types of brain functional sub-circuits (*e.g.*, dis-assortative or core-periphery) beyond the traditional assortative mesoscopic structures [11, 18]. Specifically, Sandon and Abbe, in [2], laid out a comprehensive treatment of criteria for mesoscopic structure recovery for any pair of a networked system and an *a priori* set of communities (or functional networks in brain connectomic domain). Specifically, the recovery requirements were classified under:

- Weak Recovery (also known as community detection);
- Almost Exact Recovery;
- Exact Recovery.

The recoverability of the ground-truth partition depends on the degree regime (indicated by the degree scaling factor $s_{t}$) in which the network resides. For instance, weak recovery only requires the necessary condition for a limiting graph (n→∞) to be in the constant degree regime, *i.e*., $O\left(\frac{1}{n}\right)$. On the other hand, exact recovery requires the necessary condition (for limiting graph) that the graph is asymptotically connected, *i.e*., in the degree regime of logarithmic $O\left(\frac{log(n)}{n}\right)$. The sufficient condition for all the recovery criteria is stated in the respective theorems with different proposed measures with sharp phase transitions, as seen in [2].‡ Further details on recovery theorems are located in Supplementary Information.

Here, we chose the weak-recovery requirements as guidance for whole-brain functional connectivity estimation for four reasons:

- Although Schaefer parcellations with an increasing number of nodes allow us to project some empirical insights onto their degree regime, a rigorous theoretical argument on the degree regime is not possible for any empirical graph sequence. Hence, exact recovery of an *a priori* unique ground-truth partition is not relevant in the case of brain functional connectomes;
- Even in the empirical domain, we observe that both group-average and individual FCs become disconnected (*i.e*., the number of connected components is more than 1) after a relatively small threshold value in the interval τ∈[0.2,0.3]. Theoretically, a graph sequence is required to be connected, *asymptotically*, to fulfill the requirements for *exact recovery*. On the other hand, weak-recovery (detection of mesoscopic structures) offers a more realistic and relaxed set of criteria for this particular application. This facilitates estimating a whole-brain FC that is most suitable for an *a priori* set of FNs without evaluating the number of connected components of the thresholded FC.
- Most (if not all) mesoscopic studies of brain functional sub-circuits such as [11, 18] are based on pre-defined hypotheses, *e.g*., that the brain functional sub-circuits involve a more diverse class of community than just assortative ones [11]. Such assumption leads to the appropriate usage of different community detection algorithms such as Weighted Stochastic Block Models (WSBM) in the case of [11, 18]. As mentioned above, weak-recovery is equivalent to community detection in the theoretical SBM literature;
- No set of functional sub-circuits is universally agreed and uniquely identified as the **ground-truth communities**. Hence, all proposed brain functional sub-circuit parcellations, *e.g*., [47], are relative.

### 2.2. SBM description, inference and extended usage

#### 2.2.1. Model Description

In this section, we define some of the key components of SBM. Other fundamental mathematical notations are referred to in the section Stochastic Block Model Preliminaries in Supplementary Information.

- $G=\left[a_{uv}\right]=\left\{\begin{array}{ll} FC, & \text{weighted-graphs}\\ M, & \text{binarized-graphs} \end{array}\right.$: network/graph (e.g. FCs in the context of this work);
- V(G)={u}, and E(G)={uv∣u,v∈V(G)} be set of vertices and edges, respectively;
- The size and order of a network are denoted by |V(G)|=n and |E(G)|, respectively;
- $\left\{G_{t}\right\},$ ∀t∈N is the graph sequence; in the empirical domain, the number of graphs in the sequence is defined as $\left|\left\{G_{t}\right\}\right|=T$;
- k is the number of communities/clusters;
- $\sigma =\left[\sigma_{u}\right]\in [k{]}^{n}$ is the pre-defined, well-understood community assignment in vector form of length n. It is the mathematical map σ={u↦i,∀u∈[n],i∈[k]}. In general, σ is also referred to as a graph partition;
- $\Omega =\left[\left|\Omega_{i}\right|\right]$ is the vector containing cardinality of community where
$$\left|\Omega_{i}\right|=\left|\left\{u|\sigma_{u}=i\right\}\right|,\;\forall i\in \left[k\right],u\in [n]$$
- C is the statistical summary of edge properties within and between communities in matrix form. Mathematically,
$$C=\left\{\begin{array}{l} C_{bin}\in N_{+}^{k\times k}\\ C_{wei}\in R^{k\times k} \end{array}\right.$$
  where $C_{\mathrm{bin}}\in N_{+}^{k\times k}$ denoted the simple edge count matrix within or between communities and $C_{wei}$ denoted the weighted edge sum (also within or between communities);
- $C_{\max}\in N_{+}^{k\times k}$ is the maximum number of edges within or between communities;
- $p=\left[p_{i}\right]$: the probability that a node u belongs to community i∈[k]; P=diag(p) is a k×k matrix filled with $p_{i}$ in the diagonal;
- $Q=\left[Q_{ij}\right]\in R^{k\times k}$ is the expected node degree matrix, *i.e*. the expected number of connections a node in community i has with community j;
- $s_{t}$: scalable factor of degree regime in a graph sequence $G_{t}$ where t∈T;
- $W_{s_{t}}={\left[w_{ij}\right]}_{s_{t}}$ is the edge probability between 2 nodes in community i and j in terms of the scaling factor $s_{t}$.^§^ We use W to denote the edge probability matrix with $s_{t}=1$;
- $PQ=nP\left[\frac{W}{s_{t}}\right]=nPW$ is the community profile matrix where i column is the expected number of edges that community i has with all communities. Note that for weak-recovery (detection), scaling factor $s_{t}=1$.

#### 2.2.2. Inference and extended usage

The basis of SBM parameter inference is reverse engineering by the maximum likelihood principle. Specifically, since both G and σ (subsequently, $k={max}_{u\in [n]}\sigma_{u}$) are priors, in expectation, we can infer SBM(P,W) using the Bayesian approach as follows:

- $P=\frac{\Omega }{n}=\left[p_{i}\right]=\left[\frac{\left|\Omega_{i}\right|}{n}\right]$
- Infer $W_{bin}=\frac{C_{bin}}{C_{\max}}$
- Compute $W_{wei}=\frac{C_{wei}}{C_{\max}}$
- Q=nW as $s_{t}=1$ for weak-recovery
- Compute PQ (*Matrix Multiplication*)

where $C_{bin}$ is a simple edge count of $M_{\tau }^{GA}$ between or within blocks of communities whereas $C^{\mathrm{wei}}$ is the sum of weighted edges of $FC_{\tau }$ (also between or within communities). Specifically,

$$C_{bin}=\sum_{u,v\in [n]}1_{\sigma_{u}=\sigma_{v}}$$

$$C_{wei}=\sum_{u,v\in [n]}\left|w_{uv}\right|,\;\sigma_{u}=\sigma_{v}$$

and

$$C_{max}=\Omega \Omega^{T}$$

The inference of matrix P is based on the law of large numbers [2]. For $W_{bin}$, we perform entry-wise divisions of matrix $C_{bin}$ by matrix $C_{\max}$, which infers the Bernoulli random variable parameter p representing the probability of successful edge formation between each pair of stochastically equivalent nodes within or between communities. In the case of $C_{\mathrm{wei}}$, note that we use the term **computing** instead of inferring because we have extended the usage of SNR to mesoscopic prominence measure. We use the absolute values $∥w_{uv}∥$ to only consider the overall magnitude (and not the sign) of functional couplings within/between FNs.

Technically, this inference is less challenging than traditional inference problems where σ is also a latent variable in the model and graph ensemble G is the only observable ensemble available. Specifically,

(G,n,σ,k)∼SBM(P,W)

where G and σ are priors.

### 2.3. Weak Recovery of ground-truth partition

#### Definition 1.

*Weak recovery of a ground-truth partition can be rigorously equivalent to the existence of an algorithm that infers a partition that agrees with the ground-truth one up to* ${max}_{i}p_{i}+\epsilon ,\;\forall i\in [k]$. *This level of accuracy is the minimal requirement for most community detection methods*.

#### Theorem 1.

(*Sandon and Abbe* [2]) *Let* $(G,\;\sigma )∼SBM\left(n,\;p,\;\frac{s_{t}Q}{n}\right)$ *for* p, Q *arbitrary and* $s_{t}=1$. If SNR>1, *then weak recovery is efficiently solvable; where*

$$SNR=\frac{\lambda_{2}^{2}}{\lambda_{1}}$$

and $\lambda_{i}$ is the i^th^ eigen value of the community profile matrix PQ.

Weak recovery of given ground-truth communities means that through that algorithm, the recovered partition outperforms a random guess, *i.e*. ${max}_{i}p_{i}$, by a small factor ϵ. The criteria for weak recovery are driven by a hard threshold approach presented in the below theorem. Importantly, achieving weak recovery does not necessitate the graph being connected under an asymptotic regime. Loosely speaking, we only need every graph in the graph sequence to have a large connected component. In other words, we only need $\left\{G_{t\in T}\right\}$ to be in the constant degree regime, *i.e*. $s_{t}=1$. Consider a network of n nodes divided into two equal-sized ground-truth communities (*i.e*. $\frac{n}{2}$ nodes for each community). In a weak recovery scenario, it is feasible to accurately identify the community membership of each node with a probability marginally above 50%, say by an additional 5%. It implies that if an ensemble is generated under a constant degree regime, one can arbitrarily assign any community membership to isolated nodes, *i.e*. leaves; hence, exact recovery is impossible in this regime. On the other hand, for exact recovery, since W scales with n through the factor $s_{t}$, the community profile matrix M consequently grows with the factor $s_{t}$ as well.

### 2.4. The fitness assessment framework

**Figure 1:**
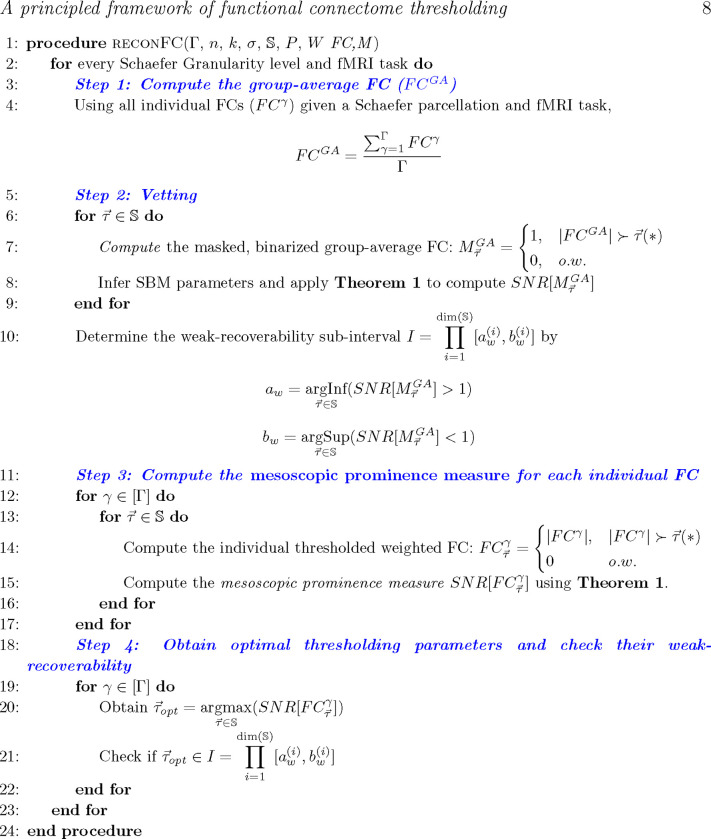
Pseudo-code for *reconFC* routine using the number of individual FCs Γ, Schaefer granularity n, number of functional networks k, *a priori* partition σ, thresholding parameter space S, community assignment likelihood P, and connectivity pattern matrix W.

For a given pair of a complex network (*e.g.*, functional connectome) and an *a priori* set of ground-truth communities (*e.g.*, Yeo’s functional sub-circuits), we propose the following steps to access the information-theoretic fitness of ground-truth communities (Figure 1) as follows:

- Step 1: Obtain an average representation (*e.g.*, a group-average FC) from the collection of individual networks, binarize the group-average representation, apply Theorem 1 across the thresholding parameter space S, and yield the masked, binarized group-average FC;
- Step 2 (Vetting Step): Compute the SNR for the masked group-average FC and investigate the SNR across all finite combinations in S. Compute the the weak-recoverability sub-interval I⊆S;
- Step 3: Compute the *a priori* community prominence for each individual FC; note that this prominence can also be computed for the group-average FC.
- Step 4: For each individual FC, obtain the $\vec{\tau }$ maximizing the the prominence computed in Step 3. Check if $\tau_{\mathrm{opt}}$ belongs to the weak-recoverability sub-interval I. Note that similar to step 3, this step can also be performed on the group-average FC.

## 3. Application: A pipeline for thresholding functional connectomes

The below pipeline describes the process to compute the optimal threshold for a given fMRI condition, a Schaefer granularity, and a cohort in two particular cases:

- individually driven threshold $\tau_{\mathrm{opt}}^{\gamma }$;
- constant (cohort-driven) threshold $\tau_{\mathrm{opt}}^{GA}$ where GA stands for group-average

Here, we see that the parameter space S reduces to the line search of threshold value τ=[0,1]∈S. The pipeline contains four distinct steps:

- Step 1: For each Schaefer granularity level and task, compute the binarized (masked) group-average FC (denoted as $M_{\tau }^{GA}$) using the entry-average of individual FCs (the number of individual FC is denoted as Γ)
- Step 2 (**Vetting Step**):
  - For each threshold value $\vec{\tau }=\tau \in [0,\;1]$, **infer** the Stochastic Block Model (SBM) parameters to compute the Signal-to-noise ratio (SNR) of $M_{\tau }^{GA}$:
$$SNR\left[M_{\tau }^{GA}\right]=\frac{\lambda_{2}^{2}}{\lambda_{1}}\left\{[PQ{]}_{bin}^{GA}\right\}$$
  - Repeat this computation for all threshold values, apply Theorem 1 to determine the weak-recoverability sub-interval $\left(a_{w},b_{w}\right)⊊\tau =[0,\;1]$ for the group-average FC, *i.e.* $M_{\tau }^{GA}$
- Step 3: For a given individual FC and threshold value τ, compute the associated thresholded FC, *i.e.*, $FC_{\tau }^{\gamma }$, and then compute the Stochastic Block Model (SBM) parameters for $FC_{\tau }^{\gamma }$. Extend the usage of SNR as a **mesoscopic prominence measure**:
$$SNR\left[FC_{\tau }^{\gamma }\right]=\frac{\lambda_{2}^{2}}{\lambda_{1}}\left\{[PQ{]}_{wei}^{\gamma }\right\}$$
  Analogously, we can also compute the SNR for the group-average FC $\left(FC^{GA}\right)$ as follows:
$$SNR[FC_{\tau }^{GA}]=\frac{\lambda_{2}^{2}}{\lambda_{1}}\left\{[PQ{]}_{wei}^{CA}\right\}$$
  Repeat step 3 for all threshold values τ∈[0,1] and all individual FCs for a given fixed Schaefer parcellation and fMRI task pair;
- Step 4:
  - Obtain the threshold value that maximizes SNR of the thresholded FC and the corresponding optimally reconstructed whole-brain FC;
$$\tau_{\mathrm{opt}}^{\gamma }=\underset{\tau }{argmax}\left(SNR\left[FC_{\tau }^{\gamma }\right]\right)$$
    Note that if the group-average FC $\left(FC^{CA}\right)$ is used in **Step 3** then:
$$\tau_{opt}^{GA}=\underset{\tau }{argmax}(SNR[FC_{\tau }^{GA}])$$
  - Check if $\tau_{\mathrm{opt}}$ is in the weak-recoverability sub-interval computed in Step 2:
$$\tau_{opt}\in \left(a_{w},{\;b}_{w}\right)$$
    Note that one needs to check the optimal threshold against the weak-recovery sub-interval, regardless of whether it is an individualized threshold ${(\tau }_{\mathrm{opt}}^{\gamma })$ or a group-average one $\left(\tau_{\mathrm{opt}}^{\mathrm{GA}}\right)$.

## 4. Results

In this section, using weak recovery criteria, we investigate the level of information-theoretic prominence of an *a priori* set of FNs with respect to different FCs (both group-average and individual subject levels) across a range of threshold values. Additionally, we offer deeper insights into the use of SNR as a measure of the information-theoretic prominence of this predetermined set of FNs. The dataset used in this paper contains 410 unrelated subjects (HCP, Q3 release). This includes (test and retest) sessions for resting state and seven fMRI tasks: gambling (GAM), relational (REL), social (SOC), working memory (WM), language processing (LANG), emotion (EMOT), and motor (MOT). Whole-brain FCs estimated from this fMRI dataset include 9 distinct Schaefer granularity levels that parcellate the cortical regions into n=100 to n=900 nodes, with a 100 nodes increment for each parcellation. The functional communities evaluated in this framework include seven cortical resting state FNs from [47]: visual (VIS), somatomotor (SM), dorsal attention (DA), ventral attention (VA), frontoparietal (FP), limbic (LIM), default mode (DMN). Each Schaefer granularity has a corresponding Yeo’s FN parcellation. Additional details about the dataset are available in Supplementary Information.

### 4.1. Weak-recoverability sub-interval $\left(a_{w},b_{w}\right)$

**Figure 3:**
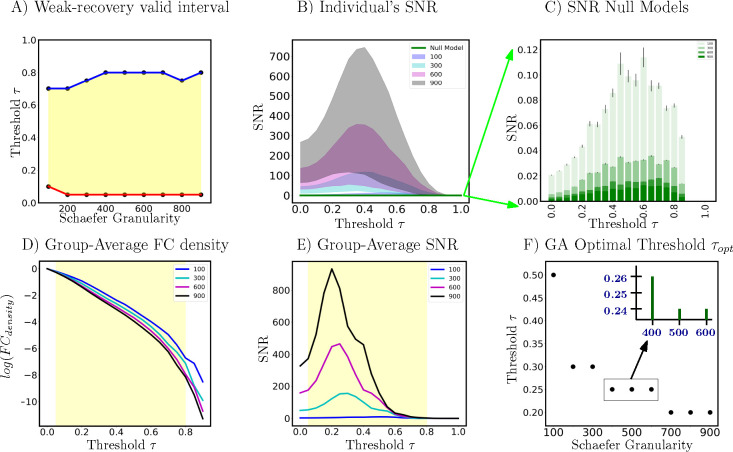
Panel (A) is the weak-recoverability sub-interval of $\tau \in \left(a_{w},\;b_{w}\right)⊊[0,\;1]$ (**Step 2**). Panel (B) is the 5- and 95- percentile of individual subjects’ SNR for four distinct Schaefer parcellations n=100,300,600,900. Panel (C) illustrates the SNR null models. Panel (D) is the FC density, on a logarithmic scale, across the same 4 granularity levels. Panel (E) shows SNR profiles computed on group-average FCs (again, over the same granularity levels). Finally, Panel (F) reports the optimal threshold $\tau_{\mathrm{opt}}$ computed based on maximum SNR of group-average FCs. Note that in panels (D) and (E) the weak-recoverability sub-intervals use the maximum and minimum values for the upper and lower bound, respectively, across Schaefer parcellations.

Based on panel (A) of figure 3, we see that for most Schaefer granularity levels (except for n=100), the lower and upper bound of theoretically guaranteed sub-interval of weak-recovery stay fairly stable: τ∈[0.05,0.8]. The lower bound $a_{w}$ stabilizes faster than the upper bound $b_{w}$, across Schaefer parcellations. Except for the low-resolution parcellation n=100, the weak-recovery valid range is relatively stable and parcellation-independent. This implies that the information-theoretic relevance of an *a priori* set of FNs is, to some extent, parcellation-free. In other words, for all investigated granularity levels, the thresholded graphs are in the weak-recoverability regime, except for the complete (τ∈[0,0.05)) or empty (τ∈(0.8,1]) graph extremes. Panel **D** of figure 3 shows further details on the FC density. This is rather interesting because, at those two extremes, networks will contain either too much noise (complete graphs) or too little signal (empty graph) for any highly putative partitions to be information-theoretically relevant.

### 4.2. Resting State: Group-Average versus Individuals

Based on panels (B) and (E) of figure 3, it is evident that all SNR profiles (including the group average and individual levels) behave non-monotonically across the threshold range. There exists a threshold value such that SNR is maximized in the investigated range τ∈[0,1]. In addition, all optimal threshold values, for both group-average and individual FCs, are within the weak-recoverability sub-interval $\left(a_{w},b_{w}\right.)$ for all investigated Schaefer granularity levels.

Secondly, we see that both group-average and individual SNR profiles scale with *n*. This is because the scaling factor $s_{t}$ for the Schaefer FC sequence is not constant. In other words, as the graph size gets larger, one can expect the community profile matrix PQ with entries $\left[PQ{]}_{ij},\;\forall i,\;j\in [k\right]$ to represent the number of expected ”friends” between FN i and j (*e.g.* between DMN and LIM) to get larger numerically. Further evidence on empirical exploration of Schaefer graph sequence degree regime is located in Supplementary Information.

Thirdly, for a fixed Schaefer granularity level, the group-average SNR peaks **higher and earlier** across the investigated threshold range than that of an individual subject. Interestingly, the topological property of *connected components* for both individual and group-average FCs, across all Schaefer parcellations, also exhibit a similar trend. Specifically, according to supplemental figure (Figure S1), individual FCs get fragmented earlier, *i.e.*, the number of connected components surpasses 1 faster, compared to the corresponding group-average FCs for a fixed granularity level. Topologically and numerically speaking, averaging FC entries across the subject domain damps down the individual fingerprints presented as high-magnitude Pearson correlation values in FCs. This results in magnitude-wise smaller functional connectivity entries, which get annihilated by smaller threshold value τ. On the other hand, using the same analogy, one can see that it takes a higher threshold value for individual FC entries to be annihilated.

### 4.3. Individualized optimal thresholds

As one can observe from figure 5, the individualized optimal threshold varies across different individuals, which demonstrates strong evidence of the existence of FN functional fingerprint across subjects. In addition, the average of these individualized thresholds, for a given parcellation granularity, is roughly equal to the group-average optimal threshold.

### 4.4. Group-average: Resting State vs. fMRI Task Analysis

Next, we investigate the prominence of Yeo’s resting state networks with respect to different fMRI conditions, including 7 tasks and the resting state, through SNR measures using group-average FCs across all Schaefer granularity levels and the entire threshold range. Using the resting state SNR profile as a baseline, we compare all task responses in these two scenarios:

- constructing FCs with the maximum number of timepoints available for each fMRI condition;
- for all fMRI conditions, constructing FCs using 166 timepoints, which correspond to the number of time points associated with the EMOTION task that is also the minimum across all conditions.

Firstly, in both scenarios, the **maximum** SNR values for all examined tasks surpass the hard threshold SNR=1 for weak recoverability. Moreover, the optimal threshold $\tau_{\mathrm{opt}}$ consistently falls within the range $\left(a_{w},b_{w}\right.)$. Trivially, resting state SNR dominates all available tasks across all parcellation levels. This is expected because the selected set of FNs are Yeo’s resting state networks. Secondly, working memory (WM) fMRI responds fairly consistently across all granularity levels in both scenarios. From an information-theoretic perspective, EMOTION is the most similar task to the resting state, with respect to Yeo’s resting state networks.

**Figure 4:**
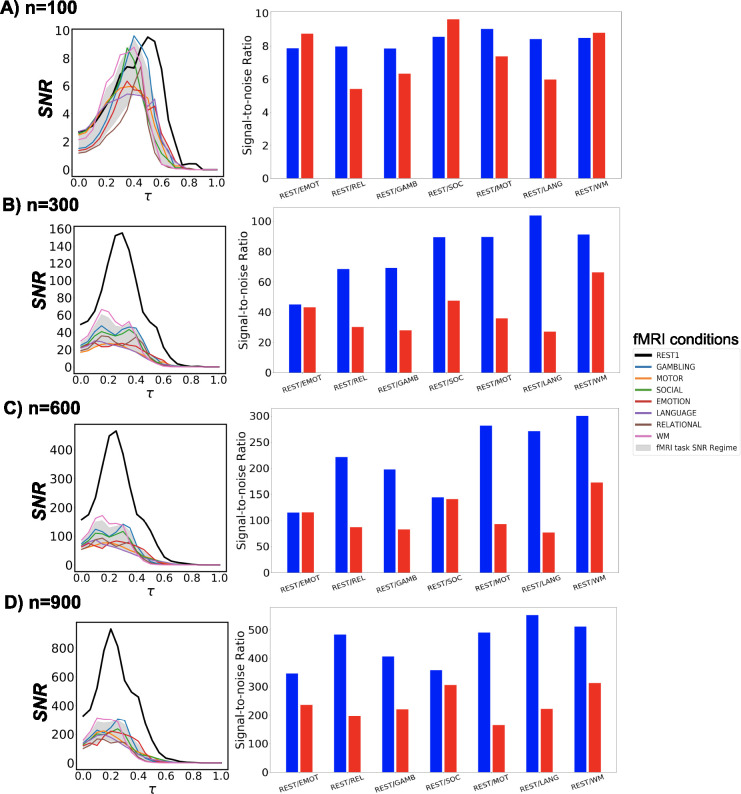
fMRI task and rest SNR profiles. For each row of panels, the left one represents SNR behavior across threshold range τ∈[0,1] using the **maximum** scanning length for all fMRI tasks and rest. The gray shade represents the 5- to 95- percentile of SNR task regime across all fMRI tasks and the entire threshold range. In the right panel, SNR profiles for task and resting-state fMRI are computed using 166 timepoints corresponding to the scanning length of the shortest scanned task, *i.e.*, EMOTION. Results are for 4 Schaefer parcellation levels: n=[100,300,600,900].

**Figure 5:**
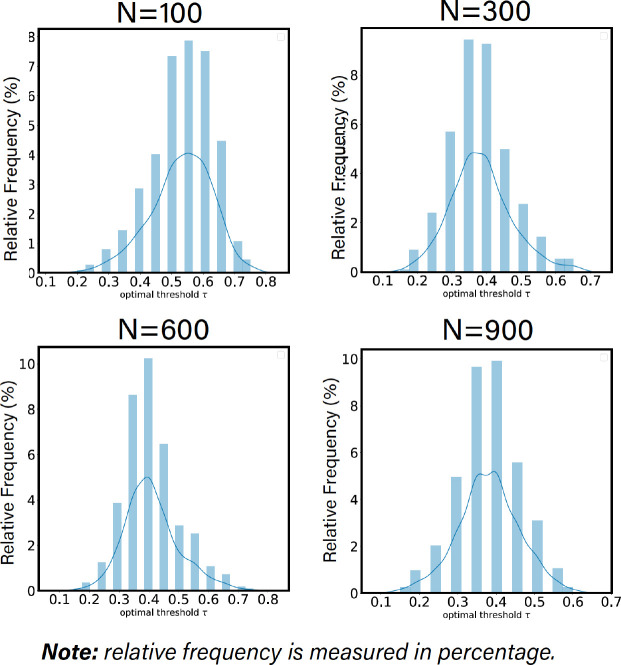
Individualized optimal threshold is derived using the SNR behavior of each individual for 4 distinct Schaefer’s parcellations n={100,300,600,900}.

Thirdly, in the maximum-timepoint case, with the exception of n=100 parcellation, the SNR profiles for most tasks are roughly half the magnitude of the resting-state SNR. Furthermore, for all examined Schaefer parcellations, group-average task FCs appear to reach their SNR peak earlier than the resting-state counterpart. Further details are indicated in figure 4 - Panel **A**.

In the second scenario when the minimum number of timepoints is used across all fMRI conditions, the gap in SNR magnitude between the resting state and each task condition is significantly narrowed, yet the SNR during rest still exceeds those during tasks.Further details are indicated in figure 4 - Panel **B**.

### 4.5. The SNR-driven inequality

It is important to check if SNRs are robust against randomness, *i.e.*, whether they are a valid factor in deciding the threshold. To do so, we randomly shuffle Yeo’s resting state networks and recompute the SNR response. We repeat the random shuffling procedure 100 times and record the results for all nine group-average FC induced by the nine Schaefer parcellations, each of which is under the $REST_{1}$ condition with scanning pattern LR. Results for RL pattern is available in Supplementary Information.

For every fixed Schaefer parcellation granularity, the null model SNR profiles are uniformly lower than those of all subjects across the entire thresholding range. Furthermore, the null model values do not exceed the hard threshold imposed by weak recovery criteria, *i.e. SNR=1*. This observation holds true across all investigated Schaefer parcellations, as seen in panel **F** of figure 3. Interestingly, the SNR gets uniformly smaller as Schaefer parcellation granularity increases, as seen also in Panel **F**.

Collectively, given the SNR results obtained at rest, under task conditions, and null models, we empirically form an inequality relation between resting state and task fMRI-induced FCs in terms of SNR response and the corresponding level of prominence of Yeo’s resting state networks across different fMRI conditions:

(1) $$0<SNR_{\mathrm{null}}<SNR_{\mathrm{task}}<SNR_{\mathrm{rest}}$$

This general order of SNR response is observed at the threshold τ that maximizes the objective function SNR by the weak-recovery criteria. At such optimal threshold values, all SNR profiles for task fMRI are in the weak recoverability region while still smaller in magnitude than that at the resting state. Together, these inequalities constitute an empirical lower-bound and upper-bound for $SNR_{\mathrm{task}}$, at least for all the tasks investigated in our study.

### 4.6. Maximum SNR and threshold relationship

As the granularity of Schaefer parcellation gets finer, the corresponding group-average SNR profiles get larger due to the natural scaling of the community profile matrix PQ. This observation applies to the majority of the threshold range. Moreover, per figure 3 Panel **F**, we see that optimal thresholds, *e.g. $\tau_{\mathrm{opt}}$*, tend to decrease as the granularity level increases, which suggests that larger Schaefer FCs do not need to be thresholded as much. Another interesting observation is that with the exceptions of n={100,200,900}, all other investigated granularity levels accept a very stable optimal threshold $\tau_{\mathrm{opt}}^{GA}=0.25$. Being a computation pipeline that relies on discretized line search on threshold τ (of increments 0.05 for τ=[0,1]), yielding this level of consistence of optimal value is unexpected.

### 4.7. Highly putative partition back-test

The theory of weak recovery and its extended usage proposed here allowed us to argue for the relevance of using SNR as a measure that, through thresholding, guides the estimation of functional connectivity given an *a priori* set of FNs. In this section, we juxtapose SNR as a guiding measure against objective-function community detection methods. One such method is Newman’s Q-score maximization [31, 32, 33]. In broad strokes, the Q score, *modularity score*, measures the statistical difference between a network and its corresponding null model with similar topological properties such as the degree sequence. It can be computed as follows:

$$Q=\sum_{u,v}\left(A_{uv}-\alpha P_{uv}\right)\delta \left(\sigma_{u},\sigma_{v}\right)$$

where δ(•,•) and α are the Kronecker delta and tuning parameter (defaulted at α=1), respectively.

In network neuroscience, the majority of studies examining mesoscopic structures of brain functions heavily leverage the maximization of Q score, which unravels predominantly assortative communities, *i.e.*, mesoscopic structures with denser internal edge density than the external one. SBM inference methods like Weighted SBM Inference, in principle, uncover a more diverse type of communities beyond assortative ones, such as dis-assortative and core-periphery communities [11]. Because of such distinct differences in principle between the two types of approaches, Q score would provide a good benchmark for comparing the robustness of SNR against various community detection approaches. Note that for Weighted SBM inference, we assume a Poisson distribution for the weighted graph [23]. Although other model assumptions are possible, our goal in this paper is not to select the most fitting model assumption but rather to investigate the differences in the communities detected using two theoretically different approaches. In other words, we are not looking to see if Q score or SNR picks up the exact threshold where the inferred partition is information-theoretically aligned with Yeo’s FNs; rather, we are interested in seeing whether each of those two measures captures the threshold interval where the two partitions agree to a relatively high degree. To measure the information-theoretic agreement between the inferred and ground-truth partitions, we use adjusted mutual information (AMI), which is a measure adjusted to chance. Further details on inference method and AMI are described in the Supplementary Information.

**Figure 7:**
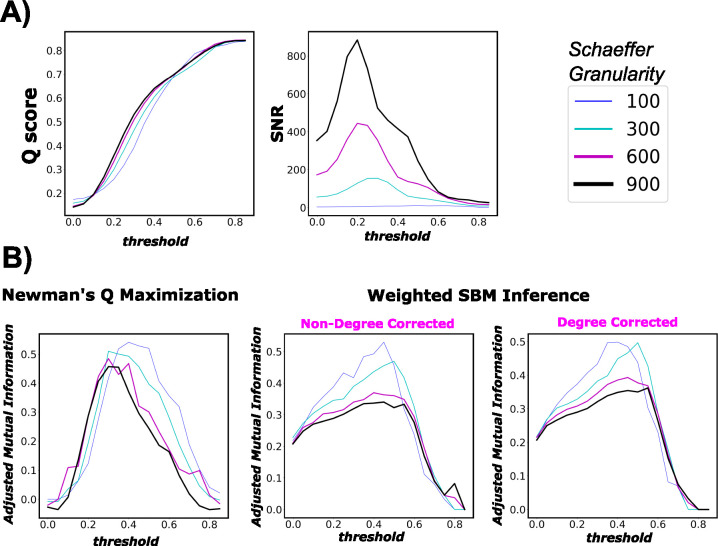
Panel (A) - left figure represents the modularity score of a thresholded group-average FC across threshold range τ∈[0,0.85]. Panel (A) - right figure reports the normalized mutual information between the inferred partition (using Q-score maximization heuristics) and Yeo’s FN partition. The same order goes to Panel (B). Panel (B) represents the results for the SNR approach. Note that the full threshold range is not necessary because in the sub-interval *τ∈[0.9,1.0]*, the thresholded graph is almost (if not) empty. The displayed result is for the group-average FCs, over four Schaefer granularity levels n=[100,300,600,900].

Firstly, per figure 7 right panels, we see that both community detection methods, namely Newman’s Q-score Maximization and Weighted SBM Inference, yield very similar trends. Specifically, both AMI profiles go up and down crossing the threshold range. Further, AMI gets smaller as n gets larger, which is expected for graphs with increasing numbers of nodes. Interestingly, the threshold values that maximize the AMI for Newman’s Q-score Maximization tend to shift left as n increases. We see this particular behavior with SNR in the earlier result section (Panel F of figure 3).

Secondly, Q score keeps a fairly steady rise in magnitude across the threshold range. Further, it does not appear that Q score is parcellation dependent; this is expected because the measure is normalized by 2m. Moreover, Q score peaks and plateaus at a very high threshold range τ∈[0.6,0.8]. In that range, the thresholded FC is highly fragmented (Figure S1) with extremely low edge density 3 and ceases to retain interesting topological insights for further analysis.

Lastly, we see that SNR driven curves, with an *a priori* set of FNs, behave very similar to AMI profiles of both objective-function approaches. On the other hand, Q score keeps rising across the threshold range and starts plateauing towards the end, which indicates failure at picking a threshold useful for an *a priori* partition such as Yeo’s FNs. Collectively, our results show, once again, that SNR computation on weighted, thresholded FCs provides excellent guidance for reconstructing a graph with the most information-theoretic relevance to a particular fixed set of FNs.

## 5. Discussion

In recent years, the network neuroscience field has been striving forward with many exciting discoveries that are becoming more and more relevant to clinical applications and personal medicine. In network neuroscience, this urges the need to improve a popular proxy of brain function, namely functional connectivity. Having the most proper, state-of-the-art mathematical representation of functional brain circuits allows for more accurate and confident positioning of research endeavors. In this work, we put forth a simple framework that allows improving the mathematical representation of brain functions given the use of an *a priori* set of functional networks. This framework also doubles as a clear evaluation tool for any specific combination of FC parcellation, FN partition, and edge pruning techniques applied to a large-scale brain dataset, thereby streamlining the complex yet crucial studies in network neuroscience.

Thresholding, which is an edge pruning technique used in post-FC processing, is seldom challenged as a standard practice that eliminates, albeit arbitrarily, statistically spurious edges. Since an increasing body of clinical research now involves FC thresholding in the data construction pipeline, careful scrutiny of thresholding is therefore imperative. We conclude that there is no single constant threshold value that is optimal across different parcellation granularity levels, such as the Schaefer ones. In particular, from coarser to finer Schaefer granularities, the optimal threshold value decreases. This result is partially observable in the behavior of matrix W across all studied Schaefer parcellations. According to figure 7, we see that for a fixed threshold value, as Schaefer granularity increases, Yeo’s functional networks behave more in an assortative manner, *i.e.*, denser internal edge density and sparser external one. We see that through a brighter diagonal and a darker off-diagonal regime of matrix W, across Schaefer parcellations with fixed threshold value τ. Information-theoretically, it means that a larger graph (in size) tends to contain more relevant information to unravel the ground-truth partition (in our study, seven Yeo’s resting state networks); hence, we do not need to threshold the FCs as deeply as the lower granularity parcellations such as n=100. This result also suggests that FC size is proportional to the level of prominence, or fitness, of the *a priori* set of FNs. Nonetheless, the exact limit of this behavior when granularity tends toward infinity is unknown, *e.g.*, whether the optimal thresholding value will reach a plateau even if the granularity increases.

Moreover, when using SNR as a goodness-of-fit measure while fitting an *a priori* set of FNs onto the FC, while no significant differences are observed between resting state and task conditions for the low-resolution parcellation (n=100), distinct differences emerge at higher resolutions. There are two ways of interpreting this result: i) *a priori* FNs exhibit a poorer fit during rest compared to task states; ii) there is an intrinsic shift of functional network dynamics at the individual level between the resting state and the task condition. Furthermore, there is also strong evidence suggesting a wide variance in the individualized thresholds across all Schaefer parcellation granularity levels. In the same vein, our results also support the concept of *individualized parcellation* suggested by the work of Salehi and colleagues [37]. While intuitive and insightful, individualized parcellation across subjects and tasks remains computationally expensive. To that end, our work offers a well-defined tool to examine the level of relevance a particular set of functional networks exhibits when mapped onto individual FCs under different conditions. In simpler terms, it allows us to, for the first time, quantify the individual differences (through information-theoretic gap) when the same atlas is mapped across cohort and/or task domains. This paves the way for alternative frameworks that build upon our work, potentially leading to task-dependent or subject-dependent parcellation methods beyond that proposed in [37].

Our work also extends the usage of the weak-recovery theorem by leveraging SNR as a goodness-of-fit measure. Specifically, our results suggest that for the majority of threshold values, the masked binarized FCs are in the regime of week recovery. However, an open question remains: when parcellated by the Schaefer atlas for a fixed individual and an fRMI condition, is the sequence of FCs in the exact recovery regime? Future studies are needed to address this information-theoretic gap between weak and exact recoverability requirements that is reflected by two measures: SNR for weak recovery and Chernoff-Hellinger distance for exact recovery. Although exact recovery is a stronger requirement, if the Schaefer graph sequence falls within the exact recovery degree regime, the mutual information between the inferred partition (through network inference and objective-based community detection methods) and the ground-truth one (*e.g.*, Yeo’s parcellations) will be theoretically higher. Furthermore, future work should address limitations in the fMRI voxel resolution, both spatial and temporal, and those in the corresponding Schaefer parcellations, particularly their impact on the SNR when fitting Yeo’s functional networks. Specifically, further investigation should be done on the effect of voxel sizes (*e.g.*, 2 mm isotropic for the HCP data set [45]) and the repetition time (*e.g.*, 720 ms for the HCP data set [45]).

Lastly, our findings highlight two important points for brain connectomics research. First, because of the existence of individual brain fingerprints [7,19], we need to pay extra attention when applying a common, fixed atlas to individual FCs. Secondly, we show that thresholding FC matrices is not only an intuitive step during FC post-processing (*e.g.*, to eliminate statistically spurious edges) but also a necessary one if we would like to use such FCs, coupled with an *a priori* set of FNs, to support any research endeavor in brain connectomics. These results suggest a promising new direction: individualized and task-dependent parcellation methods as an alternative to fixed atlases like Yeo’s.

## Supplementary Material

Supplement 1

## Figures and Tables

**Figure 2:**
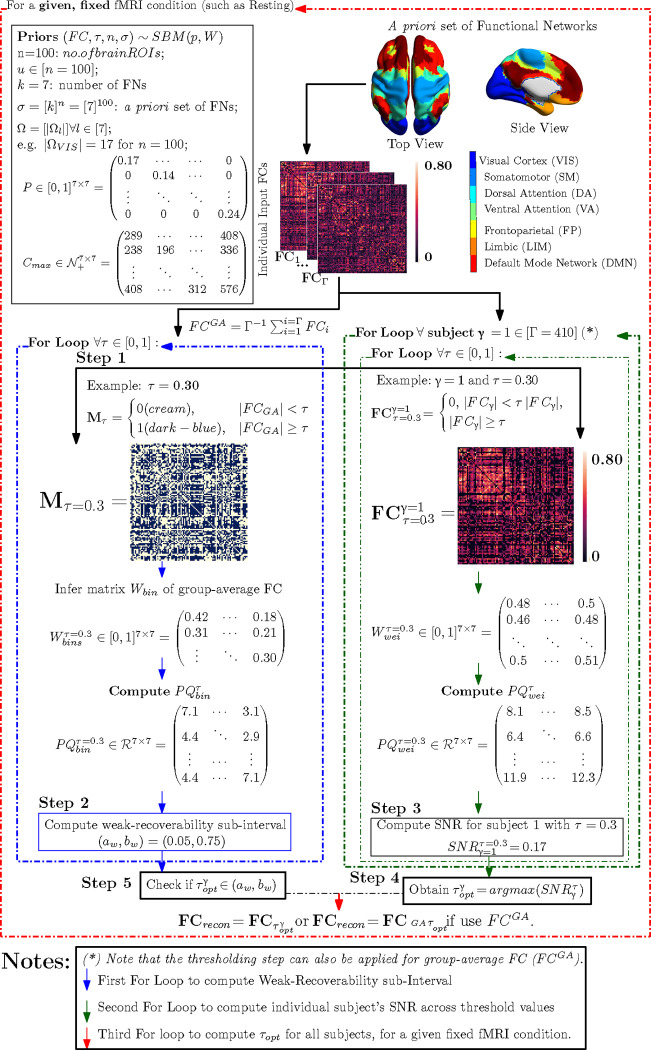
Example of the FC reconstruction routine based on the Schaefer granularity level of 100 nodes and resting-state fMRI with scanning pattern LR. Note that the *for-loop* indicated by (∗) is used to find the individualized optimal threshold for each subject, $\tau_{opt}^{\gamma }$. One can substitute this *for-loop* by finding the unique cohort optimal threshold, $\tau_{\mathrm{opt}}^{GA}$, using the group-average FC.

**Figure 6:**
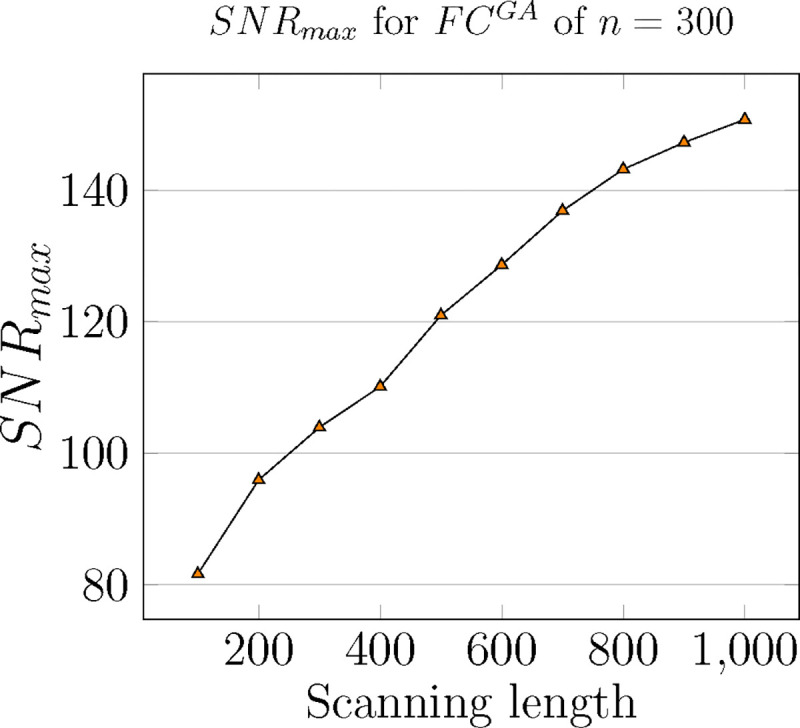
Maximum SNR computed for Resting state of Schaefer Group-average FC with n=300 for increasing scanning lengths, starting at 100 to 1000 timepoints, increments of 100 each.

## Data Availability

Data were provided [in part] by the Human Connectome Project, WU-Minn Consortium (Principal Investigators: David Van Essen and Kamil Ugurbil; 1U54MH091657) funded by the 16 NIH Institutes and Centers that support the NIH Blueprint for Neuroscience Research; and by the McDonnell Center for Systems Neuroscience at Washington University. This data used in this study are freely available on the HCP website (https://www.humanconnectome.org, accessed on 1 September 2021). The release Q3 from the HCP data with resting state and seven fMRI tasks and Glasser parcellation was used, and users must apply for permission to access the data.

## Footnotes

§ It is worth noting that if $w_{ij}$ is the same for all i,j∈[k], then SBM collapses to classical ER random graph model

‡ If a measure (say for weak or exact recovery) is below a certain algebraic threshold (stated in the respective theorems), recovery is not possible although the necessary condition is satisfied.
