# Supplementary material for: A principled framework to assess the information-theoretic fitness of brain functional sub-circuits
Source: ArXiv. 2024 Jul 23:arXiv:2406.18531v2. Originally published 2024 Jun 26. Preprint. [Version 2] (PMC11230349)
Supplement: 1 [file NIHPP2406.18531V2-supplement-1.pdf]

arXiv:2406.18531v2 [q-bio.NC] 23 Jul 2024

Supplementary information: A principled framework to assess the information-theoretic fitness of brain functional sub-circuits

Duy Duong-Tran[1,2,*,‡], Nghi Nguyen[3,‡], Shizhuo Mu[1], Jiong Chen[1], Jingxuan Bao[1], Frederick Xu[1], Sumita Garai[1], Jose Cadena-Pico[4], Alan David Kaplan[5], Tianlong Chen[6], Yize Zhao[7], Li Shen[1,†], and Joaquín Goñi[8,9,10,†]

[1] Department of Biostatistics, Epidemiology, and Informatics (DBEI), Perelman School of Medicine, University of Pennsylvania, Philadelphia, PA, USA
[2] Department of Mathematics, United States Naval Academy, Annapolis, MD, USA
[3] Gonda Multidisciplinary Brain Research Center, Bar-Ilan University, Ramat Gan, Israel
[4] Machine Learning Group, Lawrence Livermore National Laboratory, Livermore, CA, USA
[5] Computational Engineering Division, Lawrence Livermore National Laboratory, Livermore, CA, USA
[6] Department of Computer Science, The University of North Carolina at Chapel Hill
[7] School of Public Health, Yale University, New Heaven, CT, USA
[8] School of Industrial Engineering, Purdue University, West Lafayette, IN, USA
[9] Purdue Institute for Integrative Neuroscience, Purdue University, West Lafayette, IN, USA
[10] Weldon School of Biomedical Engineering, Purdue University, West Lafayette, IN, USA
* Correspondence to: duongtra@usna.edu
‡ Equal contribution
† Co-supervising Authors

The purpose of this document is to elaborate on the machinery of the morphospace and other aspects such as the dataset and brain atlas used to analyze the data. The aim is to provide further analytic results in conjunction with those already presented in the main paper.

Schaefer Sequence $\{G_{t_\ell}\}$ Topology - Resting State Analysis

Number of Connected Components

In this section, we investigate the topological features of the Schaefer FC graph sequence across the entire threshold period $\tau \in [0, 1]$. Specifically, we examine the number of components across the threshold range and Schaefer granularity levels.

We use all nine available Schaefer parcellations with $n = [100, 200, ..., 900]$ and their corresponding mappings of Yeo's seven resting state networks [17] for each granularity

level. Besides the individual level FC (denoted as FC^{γ}), the group-average FC (denoted as FC^{GA}) is computed using the entry-wise mean across the individual FCs (denoted as FC):

$$FC^{GA} = \frac{\sum_{\gamma=1}^{\Gamma} FC^{\gamma}}{\Gamma}$$

where Γ denotes the number of subjects and $\gamma \in [\Gamma]$.

Specifically, for each Schaefer granularity and threshold combination, we compute the number of components for each individual and group-average FC.

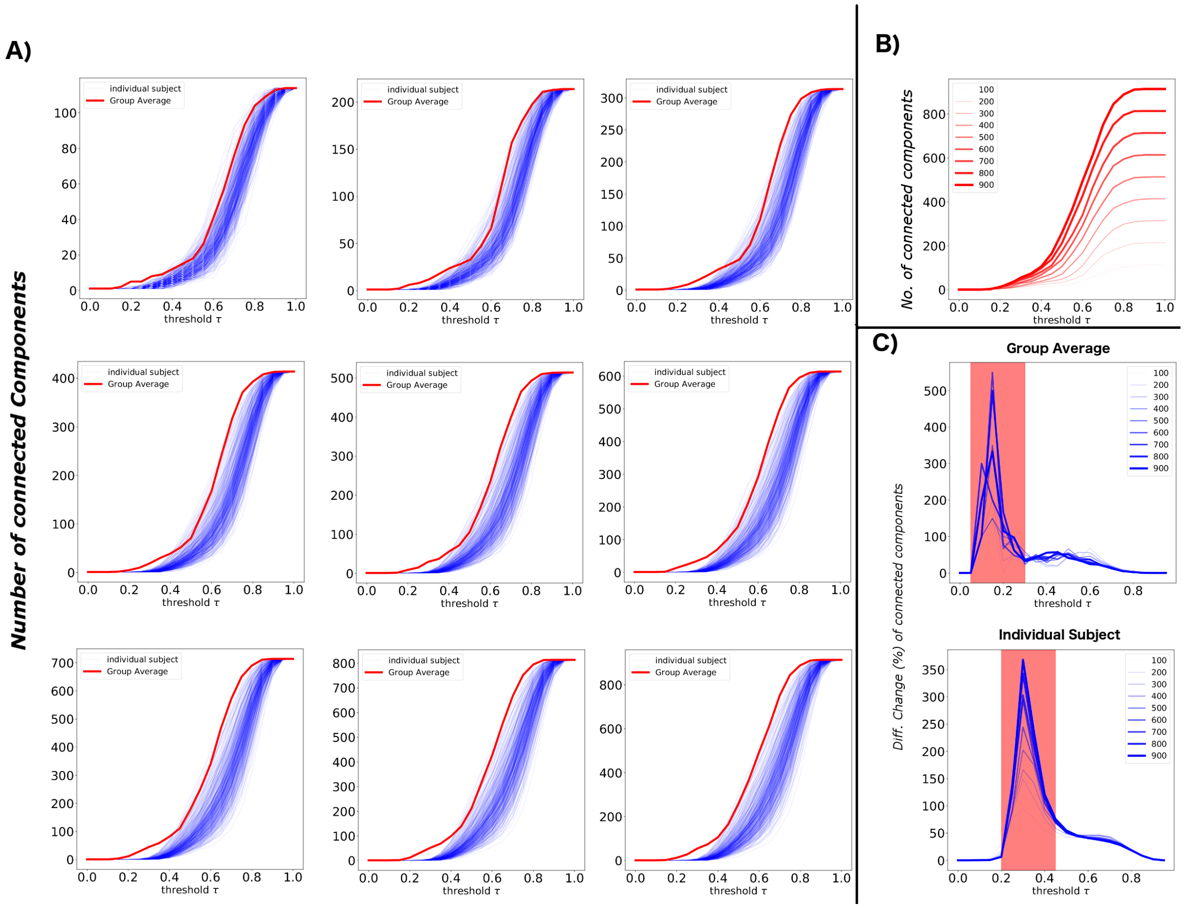

Figure S1: Panel (A) represents the number of connected components for each Schaefer parcellation (from 100 to 900 nodes with an increment of 100 nodes each time), across the pre-defined thresholding range $\tau \in [0, 1]$. Panel (B) represents the overlap in the number of components of the group-average FC for each Schaefer parcellation. Panel (C) shows the differential change (in %) between two consecutive numbers of component statistics across τ for group-average FCs (*top*) and the **mean** of individual subject FCs (*bottom*).

To study this characteristic, we use resting state fMRI data, *e.g.*, *rfMRI*. Without loss of generality, we select the first resting scan, *i.e.*, $REST_1$, with phase encoding LR. It is important to note that the connectivity (computed as the number of connected components) of the thresholded FC (where the absolute values of functional edges are set to zero - only applying step (a) above) is analogous to its binarized thresholded

counterpart (where the surviving functional edges are set to one - applying both step (a) and (b) for any given threshold and Schaefer parcellation choice). The number of components is computed using the Python package `networkx` after converting the FC matrix to a graph object.

Firstly, for all considered Schaefer parcellations, we observe that the group-average FC fragments (*e.g.*, splits into more than one connected component) earlier than the individual subjects' FCs. This is because the normalization of functional edges across the cohort domain neutralizes individual differences and zeroes out relatively faster across the thresholding range (Panel A). Moreover, it is also expected that the group-average number of connected components increases proportionally with the parcellation sizes, and for a fixed threshold value, the number of connected components in a coarser parcellation is always smaller than in a finer one (Panel B).

We also compute the differential change $\Delta\mathbb{C}$ (in percentage) between two consecutive $\mathbb{C}$s across the threshold range as follows:

$$\Delta\mathbb{C}_l(\%) = \frac{|\mathbb{C}_{l+1} - \mathbb{C}_l|}{\mathbb{C}_l} \times 100$$

where l is indexed over the threshold range. We observe that both the group-average (Panel C-*top*) and individual level (Panel C-*bottom*) show an empirical phase transition in the number of connected components. This phase transition occurs in the sub-intervals $(0.05, 0.30)$ and $(0.20, 0.45)$ for the group-average and individual levels, respectively. Although there is a numerical overlap between the two phases, the group-average transitions earlier than the individual one.

FN-Differential Identifiability $\mathbb{I}_{diff}$ and Empirical Schaefer Degree Regime

In this section, we investigate the behavior of the matrix W_{bin} of group-average FCs, using Yeo's 7 resting state networks [17]. To make some empirical observations about the Schaefer FC sequence degree regime, we examine the group-average masked FC, M^{GA}, across all nine granularity levels and the threshold interval $\tau \in [0, 1]$ with an increment of 0.05. The reason we look only at the masked (binarized) FCs is because

- The Sandon et al. [1] theorem on weak-recovery is written for binary graphs. Hence, the recoverability requirement on the degree-regime is only applied to the binary scaffold.
- We see that looking at weighted graphs is not appropriate in this case as the row (or column) sum of the FC matrix would yield the connectivity strength of a node, not its degree.

Here, we investigate the empirical degree regime of the Schaefer graph sequence based on the behavior of W_{bin}. For all studied Schaefer granularity levels and threshold combinations, to infer W_{bin}, we simply use the maximum likelihood rule as mentioned in the main text. Recall that matrix $W_{bin} = [w_{ij}]$, where w_{ij} contains the probability that

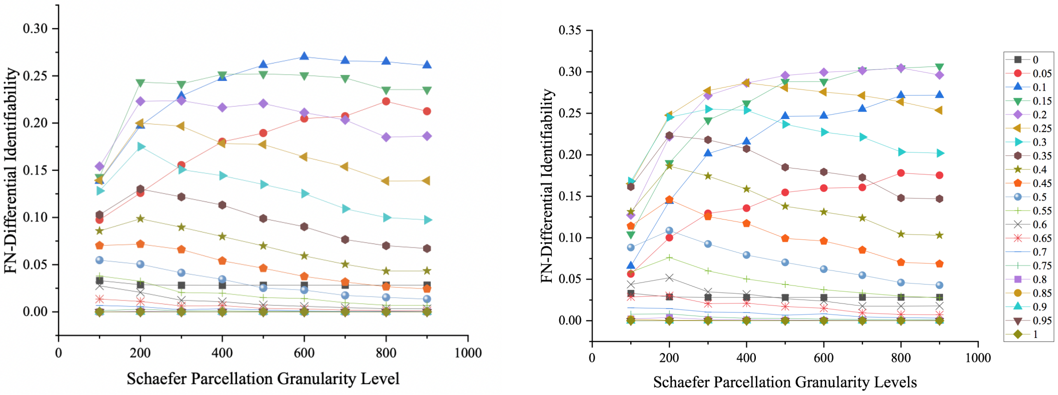

(a) FN-Differential Identifiability $\mathbb{I}_{diff}^{FNs}$ score for Language fMRI task (LR scanning pattern).

(b) FN-Differential Identifiability $\mathbb{I}_{diff}^{FNs}$ for resting state (LR scanning pattern).

Figure S2: FN-Differential Identifiability $\mathbb{I}_{diff}$ for resting state and one particular fMRI task across all threshold and Schaefer Granularity Level combinations.

a node u in community i is connected ($e.g.$, $a_{uv} = 1$) or not-connected ($e.g.$, $a_{uv} = 0$) to another node v in community j. Its entries are bounded between 0 and 1. Also, recall that in the previous section on the degree regime, the graph sequence is in a constant degree regime if the corresponding matrix W does not scale with n, $e.g.$, $s_t = 1$.

Here, we look at the behavior of the degree regime through a proposed measure, called FN-differential identifiability, inspired by Amico et al. [3], as follows:

$$\mathbb{I}_{diff}^{FNs} = \mathbb{I}_{self}^{FNs} - \mathbb{I}_{others}^{FNs} \tag{1}$$

$$= \langle W_{ii} \rangle - \langle W_{ij} \rangle \tag{2}$$

where $i, j \in [k]$ and $k = 7$ in our study. Moreover, $\langle W_{ii} \rangle$ and $\langle W_{ij} \rangle$ are the averages of the diagonal and off-diagonal entries of matrix W, respectively. We formally define $\langle W_{ii} \rangle$ and $\langle W_{ij} \rangle$ to be the differential identifiability within ($e.g.$, $\mathbb{I}_{self}^{FNs}$) and between ($e.g.$, $\mathbb{I}_{others}^{FNs}$) FNs.

Per Figure S2, for most threshold values (with the exception of $\tau = 1$), there is an intensity shift in W from between-FN connectivity to within-FN connectivity strength as $\mathbb{I}_{diff}^{FNs}$ increases across Schaefer parcellation granularity levels. In other words, within-FN identifiability $\mathbb{I}_{self}^{FNs}$ increases as between-FN identifiability $\mathbb{I}_{others}^{FNs}$ decreases. Moreover, the monotonic increase of $\mathbb{I}_{diff}$ also suggests that finer-grain Schaefer parcellations might reflect a higher level of information-theoretic prominence of the *a priori* set of FNs, given the group-average FCs. These results indicate that the Schaefer brain graph sequence resides in the diverging degree regime, with the exception of the trivial case $\tau = 1$. Empirically, these results suggest that the Schaefer graph sequence is at least not in the constant degree regime, $i.e.$, $s_t \neq 1$.

Weak Recovery - fMRI Resting State Further Analysis

Null models

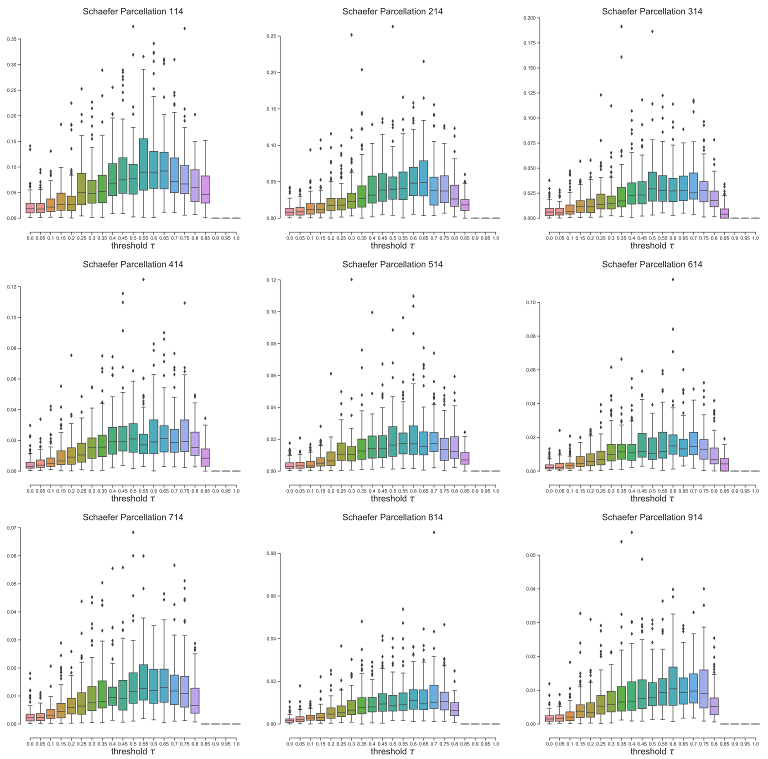

Figure S3: Each subplot represents the SNR profiles corresponding to 100 randomized parcellations for each thresholding value $\tau \in [0, 1]$ for all nine Schaefer parcellations.

The null model is assessed by feeding a randomized partition that respects Yeo's FNs sizes. The number of simulations is 100, and the scanning session is LR. Results on the empirical distribution of randomized SNR scores are shown in Figure S3.

Methods

Notations

In this section, we describe some stochastic block model (SBM) fundamentals, along with some fundamental mathematical notions that are not included in the main text. For instance, a number (scalar) is denoted with a regular letter such as x, y; a default vector is denoted by a bold regular letter *i.e.*, $\mathbf{x}$, sometimes with or without a subscripted index, and is in column format. Matrices are denoted by bold, capitalized letters, *i.e.*,

$\mathbf{A}$, while a set is denoted by a capitalized letter, *i.e.*, S. Further, $\mathbb{N}$ and $\mathbb{R}$ are sets of natural and real numbers, respectively; $[l]$ denotes all positive integers between 1 up to l. All other standard mathematical notations are assumed unless otherwise specified.

SBM Definition

The Stochastic Block Model (SBM) has a history of both depth and breadth, spanning across multiple disciplines. Here, we only extract relevant information regarding SBM literature relevant to our exploration. Stochastic Block Models (SBMs) are random graph models that generate ensembles with clusters. Specifically, they generate Erdos-Renyi (ER) subgraphs union with multi-partite graphs between those subgraphs. A traditional SBM generates binary graphs, *i.e.*, networks with $\{0, 1\}$ edges. Nonetheless, there are SBM models developed for weighted networks, which are called weighted SBM (or WSBM).

SBM Inference and Synthesis

(Binary) SBM. Since SBM is a generative model, it is essential to discuss how to synthesize ensembles using such models, *e.g.*, network synthesis, and how to infer SBM parameters using the observable ensembles, *e.g.*, network inference. In the context of our problem, we have a slightly different starting point as the partition is not latent, though generally, partitions are often inferred. Networks with an existing ground-truth partition are very rare; furthermore, those ground-truths cannot be defined in an absolute sense. The majority of SBMs are defined as follows:

$$G \sim SBM(k, p, W, \sigma)$$

However, in the context of our paper, the partition is not latent. Specifically,

$$(G, \sigma, k) \sim SBM(p, W)$$

for our application.

In the case of $G \sim SBM(k, p, W, \sigma)$, SBM seeks a partition that divides network G into k communities. The probability that two nodes are connected to each other is governed by the probability W_{σ_u,σ_v}. To fit SBM onto a network, one needs to estimate $W = [w_{ij}], \forall i, j \in [k]$ (meaning that k is an *a priori* condition for fitting) along with the community label $\sigma_u, \forall u \in [n]$. Assuming that each edge is drawn independently from identical distributions, the probability that a network $G = A = [a_{uv}]$ is generated (synthesized) from *a priori* W and σ (prior beliefs) is as follows:

$$\mathbb{P}(A \mid W, \sigma) = \prod_{u>v} W_{\sigma_u,\sigma_v}^{a_{uv}} (1 - W_{\sigma_u,\sigma_v})^{1-a_{uv}}$$

for symmetric networks. From the inference standpoint, the Bayesian posterior probability can be computed as follows:

$$\mathbb{P}(\sigma \mid A) = \frac{\sum_W \mathbb{P}(A \mid W, \sigma)\mathbb{P}(W, \sigma)}{\mathbb{P}(A)}$$

where $\mathbb{P}(W, \sigma)$ represents Bayesian prior beliefs. If there is only one W (*hard constraint*, Piexoto) that is comparable to network A and partition σ, then we can drop the summation notion, resulting in:

$$\begin{aligned}\mathbb{P}(\sigma \mid A) &= \frac{\mathbb{P}(A \mid W, \sigma)\mathbb{P}(W, \sigma)}{\mathbb{P}(A)} \\ &= \frac{\exp\{-\ln(\mathbb{P}(A \mid W, \sigma)) - \ln(\mathbb{P}(W, \sigma))\}}{\mathbb{P}(A)}\end{aligned}$$

The hard constraint assumption is a very standard technique to isolate the eventual partition σ for inference purposes. Note that the adjacency structure A is, of course, "hard" (there is only one ensemble A).

Since $\mathbb{P}(A)$ is also fixed, maximization of posterior probability $\mathbb{P}(\sigma \mid A)$ is equivalent to maximizing

$$-\ln(\mathbb{P}(A \mid W, \sigma)) - \ln(\mathbb{P}(W, \sigma))$$

which is also understood as the minimization of the description length (DL, measured *in bits*) of ensemble A using partition σ. Once again, the hard constraint assumption yields that the description length ultimately only depends on:

$$DL = -\ln(\mathbb{P}(A \mid W, \sigma))$$

In the binary SBM case, it follows that:

$$\begin{aligned}DL &= -\ln(\prod_{u>v} W_{\sigma_u,\sigma_v}^{a_{uv}}(1 - W_{\sigma_u,\sigma_v})^{1-a_{uv}}) \\ &= -\sum_{u>v} a_{uv}\ln(W_{\sigma_u,\sigma_v}) + (1 - a_{uv})\ln(1 - W_{\sigma_u,\sigma_v})\end{aligned}$$

Hence, minimization of DL is equivalent to maximizing the log-likelihood function.

Weighted SBMs. The assumption of binary edges could be unfitting for some applications, including functional brain networks where there is a need to express different levels of functional coupling strength numerically. In such cases, we need to introduce the structure of covariates (denoted as $x = [x_{\sigma_u,\sigma_v}]$) to model the weights. In this case, the prior is written as follows:

$$\mathbb{P}(x, A \mid \sigma) = \mathbb{P}(x \mid A, \sigma)\mathbb{P}(A, \sigma)$$

It follows that the posterior probability becomes:

$$\mathbb{P}(\sigma \mid A, x) = \frac{\mathbb{P}(A \mid x, \sigma)\mathbb{P}(x, \sigma)}{\mathbb{P}(A)}$$

Using a similar technique in the binary case, one can estimate the covariate structure first so that joint probabilities with x, *e.g.*, $\mathbb{P}(x, \sigma)$ and $\mathbb{P}(A, x)$, do not alter the posterior distribution behavior. Hence, the posterior belief is proportional to the priors, which can be written as follows:

$$\mathbb{P}(\sigma \mid A, x) \sim \mathbb{P}(A \mid x, \sigma)$$

Ultimately, the task of finding the "ground-truth" partition σ depends on the likelihood of prior beliefs. This is equivalent to maximizing $\mathbb{P}(A \mid x, \sigma)$. The first task is, of course, to estimate x as A is already available. We notate the covariate structure x to be more integrated with probability distribution parameter notations $\mathbb{P}(X = x)$. Specifically, we assume that the realized FC edge weights are drawn from some distributions with specific parameter(s).

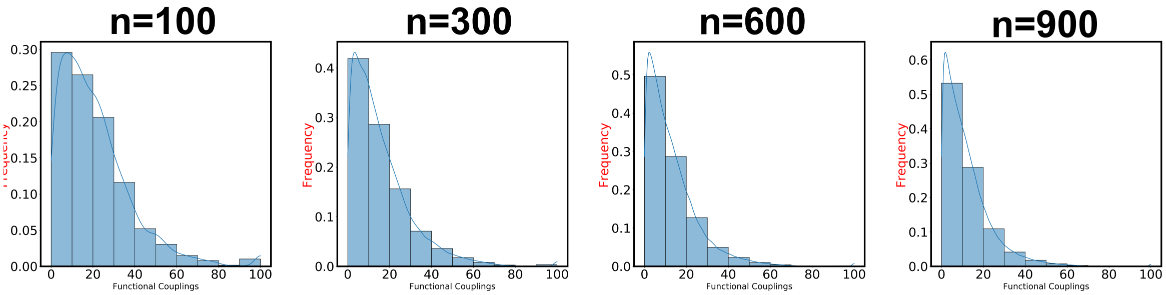

Figure S4: Empirical distribution of functional coupling magnitudes (shown as percentage points by multiplying absolute Pearson correlation values by 100) from group-average FCs across four distinct Schaefer parcellations $n = [100, 300, 600, 900]$.

Model Selection. There are different approaches to model selection (*e.g.*, which edge weight distribution one should use, given the empirical data). In the context of this paper, given the empirical distribution of FC edge weight and the usage of absolute functional connectivity (non-negative pair-wise edges), we shortlist two candidate distributions: exponential (continuous) and Poisson (discrete counterpart). Each choice has its pros and cons. For instance, choosing the exponential distribution allows us to stick with continuous ensembles of functional edge weights, which is consistent with how FC edges are computed using Pearson correlations. However, in a continuous distribution, the probability of an FC edge taking on a particular value is zero by definition. Yet, the functional connectome is sparse [5], *e.g.*, the majority of pairwise interactions between two brain regions are non-existent. Hence, using the exponential distribution will not suffice. A common approach is to use a different distribution such as the Binomial to model edge (non-)existence and connect the two distributions using a weighted average (see the methods section in [5] for further details). This will force the modeler to make a precursor assumption on weight value, which is not ideal.

On the other hand, using the Poisson distribution (a discrete counterpart of the exponential distribution) offers us the distinct advantage of modeling a non-zero probability of getting zero-valued functional edges. Recall that the Poisson probability density function is as follows:

$$f(k, \lambda) = \mathbb{P}(X = k) = \frac{\lambda^k e^{-\lambda}}{k!}$$

Clearly, $\mathbb{P}(X = 0) = e^{-\lambda} > 0$, which ultimately depends on λ inference based on empirical observations of functional edges. Nonetheless, the shortcoming of using a

discrete distribution is precisely the advantage of using the exponential one: being able to model edges in a continuous manner. To overcome this shortcoming of discrete distribution usage, we convert functional couplings (computed by Pearson correlations, which are nicely bounded between $[-1, 1]$) to percentage points and round to the nearest integer. For instance, if a functional edge has a value of $a_{uv} = 0.588$, the weighted graph will take $a_{uv} = 59$. The reason for rounding to the nearest integer is that the Poisson distribution takes on non-negative integer values $\mathbb{N}^+$. Note that using the Poisson Distribution makes no essential topological changes to the original FC other than rounding functional couplings to the nearest integer.

In this paper, we use the Poisson distribution for degree sequence as proposed by Karrer and Newman [8]. In the next sections, we review the inference procedure (as proposed in [8]) for both assumptions:

- Non-degree-corrected WSBM;
- Degree-Corrected WSBM.

WSBM Inference Procedure: In this paper, we use the method described at `https://graph-tool.skewed.de/` by Tiago Peixoto. Further treatments on weighted SBM can be found in [10]. After reviewing the inference approaches, we compare the philosophical similarities and differences between WSBM inference and Q score modularity.

Standard (Non-degree-corrected) WSBM The review of non-degree-corrected (NDC) WSBM is provided in a well-cited paper by Karrer and Newman [8]. The authors assumed such a distribution for multi-graph ensembles, where edges can take on integer values larger than 1. In this case, the prior probability can be written as follows:

$$\mathbb{P}(A \mid x, \sigma) = \prod_{u<v} \frac{(x_{\sigma_u,\sigma_v})^{A_{uv}} e^{-x_{\sigma_u,\sigma_v}}}{A_{uv}!} \times \prod_{u} \frac{(x_{\sigma_u,\sigma_u})^{A_{uu}/2} e^{-x_{\sigma_u,\sigma_u}}}{(A_{uu}/2)!}$$

It is important to note that the expected adjacency structure in this case is

$$\mathbb{E}(A_{NDC}) = YxY^{\top}$$

where $Y \in [0,1]^{n \times k}$ is the node community membership matrix, *i.e.*, $y_{ul} = 1$ if and only if node u is in community $l \in [k]$.

Note that self-loop edge weight cannot be counted twice. For symmetric networks where $A_{uv} = A_{vu}$ and $x_{ij} = x_{ji}$, the above prior probability can be written as follows:

$$\mathbb{P}(A \mid x, \sigma) = \frac{\prod_{ij} x_{ij}^{C_{ij}/2} \exp(-\frac{1}{2}|\Omega_i||\Omega_j| x_{ij})}{\prod_{u<v}(A_{uv}! \prod_u 2^{A_{uu}/2}(A_{uu}/2)!)}$$

where $|\Omega_i|$ is the cardinality of community i, C_{ij} is the counted number of edges between community i and j which can be simply computed by:

$$C_{ij} = \sum_{u,v} A_{uv}\delta_{\sigma_u,i}\delta_{\sigma_v,j}$$

where δ is the Kronecker delta function as defined in the main text. Similar to the binary case, the log-function is then be:

$$\log \mathbb{P}(A \mid x, \sigma) = \sum_{ij}(C_{ij}\log(x_{ij}) - |\Omega_i||\Omega_j|x_{ij}) + \Theta(G)$$

where $\Theta(G)$ is the quantity dependent on ensemble G (such as $|\Omega_i|$ or A_{uv}) which has no impact onto the logarithmic function behavior (*i.e.* not impacting the optimal value of this function). The inference process reduces to maximizing:

$$L(x, \sigma) = \sum_{ij}(C_{ij}\log(x_{ij}) - |\Omega_i||\Omega_j|x_{ij})$$

Note that here, we drop A (ensemble adjacency structure) just to ease notation usage and emphasize which variable(s) the likelihood function depends on. To optimize the above function, one can use differential calculus as follows:

$$\frac{dL}{dx_{ij}} = L^{'}_{ij} = \frac{d}{dx_{ij}}\left[\frac{C_{ij}}{x_{ij}} - |\Omega_i||\Omega_j|\right]$$

Setting the first derivative to zero, *e.g.* $L' = 0$, we obtain:

$$\hat{x}_{ij} = \frac{C_{ij}}{|\Omega_i||\Omega_j|}$$

Note that now we have estimated x, *i.e.*, the covariate structure, the likelihood function can be written as follows:

$$L(\hat{x}, \sigma) = \sum_{ij}(C_{ij}\log(x_{ij})) - 2m$$

Dropping the constant $2m$ (ensemble node's degree sum) and substituting the estimated covariate $\hat{x}$, the log-likelihood function can now be written as:

$$L(\sigma) = \sum_{ij} C_{ij}\log\left(\frac{C_{ij}}{|\Omega_i||\Omega_j|}\right)$$

Using simple algebra, the log-likelihood function can be rewritten as:

$$\begin{aligned} L(\sigma) &= 2m\sum_{ij}\frac{C_{ij}}{2m}\left[\log\left(\frac{C_{ij}/2m}{|\Omega_i||\Omega_j|/n^2}\right) - \log\left(\frac{n^2}{2m}\right)\right] \\ &= \sum_{ij}\frac{C_{ij}}{2m}\log\left(\frac{C_{ij}/2m}{|\Omega_i||\Omega_j|/n^2}\right) + \Theta(G) \end{aligned}$$

where, again, Θ is a constant function based on ensemble G.

Let Y and Z be the random variables representing community assignment on one end of a stub (half-edge). Then we can build a joint probability distribution between Y' and Z' as follows:

$$\mathbb{P}_\sigma = \mathbb{P}_\sigma(Y', Z') = \frac{C_{ij}}{2m}, \quad \forall i, j \in [k]$$

On the other hand, the randomized counterpart distribution of these random variables (with the same *a priori* partition σ) is

$$\mathbb{P}_{\text{null}}^{WSBM} = \frac{|\Omega_i||\Omega_j|}{n^2}$$

In this case, edge formation (from two stubs) is completely random with probability $\frac{|\Omega_i|}{n}$ and $\frac{|\Omega_j|}{n}$ for each stub. Overall, the likelihood function becomes:

$$\begin{aligned} L(\sigma) &= \sum_{ij} \mathbb{P}_\sigma(ij) \log \left\{ \frac{\mathbb{P}_\sigma(ij)}{\mathbb{P}_{\text{null}}^{WSBM}(ij)} \right\} \\ &= \sum_{ij} \mathbb{P}_\sigma(ij) \left\{ \log(\mathbb{P}_\sigma(ij)) - \log(\mathbb{P}_{\text{null}}^{WSBM}(ij)) \right\} \end{aligned}$$

On the other hand, the Kullback-Leibler Divergence between two probability distributions $P(x)$ and $Q(x)$ is defined to be:

$$D_{KL}(P \mid\mid Q) = \sum_{x \in \mathcal{X}} P(x) \log \left\{ \frac{P(x)}{Q(x)} \right\}$$

where $x \in \mathcal{X}$ is the random variable taking on values in the sample space $\mathcal{X}$. Then the log-likelihood function above can be thought of as an information theoretic measurement between the "ground-truth" probability distribution $x(\sigma)$ and the corresponding null distribution $x(\text{null})$. If we only look at what happens within communities, the quality function becomes:

$$L_{\text{within}}(\sigma) = \sum_{i} \mathbb{P}_\sigma(ii) \left\{ \log(\mathbb{P}_\sigma(ii)) - \log(\mathbb{P}_{\text{null}}^{WSBM}(ii)) \right\}$$

If we substitute the estimated $\mathbb{P}_\sigma$ and $\mathbb{P}_{\text{null}}$ above into this equation, we obtain:

$$L_{\text{within}}(\sigma) = \sum_{i} \frac{C_{ii}}{2m} \left\{ \log\left(\frac{C_{ii}}{2m}\right) - \log\left(\frac{|\Omega_i|^2}{n^2}\right) \right\}$$

Of course, we also have the log-function describing the differential information description requirement between communities:

$$L_{\text{between}}(\sigma) = \sum_{i \neq j} \frac{C_{ij}}{2m} \left\{ \log\left(\frac{C_{ii}}{2m}\right) - \log\left(\frac{|\Omega_i||\Omega_j|}{n^2}\right) \right\}$$

We will compare the within-community L_{within} with the modularity function Q score in the subsequent sections.

Degree-corrected WSBM For the degree-corrected (DC) WSBM case, a new hyper-parameter is introduced into the model θ_r (arbitrary constant terms that are $o(x_{\sigma_r,\sigma_s})$, i.e., constant terms that get absorbed into x_{ij}). The prior probability can now be written as follows:

$$\mathbb{P}(A \mid \theta, x, \sigma) = \prod_{u<v} \frac{(\theta_u \theta_v x_{\sigma_u,\sigma_v})^{A_{uv}} \exp(-\theta_u \theta_v x_{\sigma_u,\sigma_v})}{A_{uv}!} \times \prod_{u} \frac{(\theta_u^2 x_{\sigma_u,\sigma_u})^{A_{uu}/2} \exp(-\theta_u^2 x_{\sigma_u,\sigma_u})}{(A_{uu}/2)!}$$

where $\sum_u \theta_u \delta_{\sigma_u,i} = 1$ (with δ as the Kronecker delta function as usual). Basically, θ_u represents the probability that a half-edge (stub) in community i originated from u itself, where $\sigma_u = i$. It is noteworthy that the expected value of the adjacency structure in this case is no longer just x_{σ_u,σ_v} but instead:

$$\begin{aligned}\mathbb{E}(A_{DC}) = [\mathbb{E}(a_{uv})] &= \theta_u x_{\sigma_u,\sigma_v} \theta_v \\ &= \mathrm{diag}(\theta) Y x Y^T \mathrm{diag}(\theta)\end{aligned}$$

where $\mathrm{diag}(\theta) = \mathrm{diag}([\theta_u])$ is the diagonal matrix containing the θ_u weights of node u. The priors can then be condensed as follows:

$$\mathbb{P}(A \mid \theta, x, \sigma) = \frac{\prod_u \theta_u^{d_u} \prod_{ij} x_{ij}^{C_{ij}/2} \exp(-\frac{1}{2} x_{ij})}{\prod_{u<v} A_{uv}! \prod_u 2^{A_{uu}/2} (A_{uu}/2)!}$$

with d_u being the degree of node u. The log-likelihood function is then

$$\begin{aligned} L &= \log \mathbb{P}(A \mid \theta, x, \sigma) \\ &= 2 \sum_u d_u \log \theta_u + \sum_{ij} \{C_{ij} \log x_{ij} - x_{ij}\} \\ &= L_1 + L_2 \end{aligned}$$

where d_u is the degree of node u, and again, constant terms $\Theta(G)$, which contain A_{uv} terms, are ignored. The goal is to maximize this log-function, compartmentally, with respect to the normalization condition $\sum_u \theta_u \delta_{\sigma_u,i} = 1$. We look at them separately (again, ignoring any constants). Maximizing $L_2 = \sum_{ij} \{C_{ij} \log x_{ij} - x_{ij}\}$ is straightforward by taking the derivative with respect to x_{ij}. Specifically,

$$L_2' = \frac{dL_2}{dx_{ij}} = \frac{C_{ij}}{x_{ij}} - 1 = 0 \rightarrow \hat{x}_{ij} = C_{ij}$$

$$L_1 = \sum_u d_u \log \theta_u = \sum_i d_u \delta_{\sigma_u,i} \log \theta_u$$
$$= \sum_i \left\{ \sum_{u|\sigma_u=i} d_u \log \theta_u \right\} \quad s.t. \quad \sum_{u|\sigma_u=i} \theta_u = 1$$
$$= \sum_i \left\{ s_i \sum_{u|\sigma_u=i} \frac{d_u}{s_i} \log \theta_u \right\} \quad s.t. \quad \sum_{u|\sigma_u=i} \theta_u = 1$$

where $s_i = \sum_{u|\sigma_u=i} d_u$ is the number of half-edges in community i. Note that there are $|\Omega_i|$ terms of θ_u for each community. We see that L_1 is the entropy of the probability distribution representing the random variable θ, i.e., the probability that an edge in community i lands on u for which $\sigma_u = i, \forall i$. This entropy is minimized when

$$\hat{\theta}_u = \frac{d_u}{\sum_u d_u}$$

Here, it is important to note that if we choose a random uniform distribution for the random variable θ (e.g., $\hat{\theta}_u = \frac{1}{|\Omega_i|}$), we obtain minimized L_1, which reduces L.

Difference between Non-degree-corrected and Degree-corrected Models

Plugging in the estimated parameters for both cases of WSBM, we obtain:

$$L_{NDC} = \sum_{ij} C_{ij} \log \left[\frac{C_{ij}}{|\Omega_i||\Omega_j|} \right]$$

and

$$L_{DC} = \sum_{ij} C_{ij} \log \left[\frac{C_{ij}}{s_i s_j} \right]$$
$$= \sum_{ij} \frac{C_{ij}}{2m} \log \left[\frac{C_{ij}/2m}{(s_i/2m)(s_j/2m)} \right]$$

which is the Kullback-Leibler divergence between $P(x)$ (same as in the NDC case) and $Q_{DC}(x)$. In other words,

$$\mathbb{P}_{null}^{WSBM} = \frac{s_i s_j}{(2m)^2}$$

for the DC case. Recall that for the NDC case, the null model is:

$$\mathbb{P}_{null}^{WSBM} = \frac{|\Omega_i||\Omega_j|}{n^2}$$

Thus, the best fit to the NDC WSBM is the partition that most surprises the Erdos-Reyni random counterpart while for the DC WSBM case, it is the group assignment that is most surprising to the random model with the same empirical degree sequence.

Modularity

The Q-score for a given partition can be computed as follows:

$$Q(\sigma, \alpha = 1) = Q(\sigma) = \sum_{u,v} (A_{uv} - \alpha P_{uv}) \delta(\sigma_u, \sigma_v) = \frac{1}{2m} \sum_{uv} \left\{ A_{uv} - \frac{d_u d_v}{2m} \right\} \delta(\sigma_u, \sigma_v)$$

where $2m$ and d_u represent the graph and node degrees, respectively:

$$\forall u \in V(G) : d_u = \sum_{v} A_{uv} \quad \& \quad 2m = \sum_{u} d_u$$

and the default scaling factor $\alpha = 1$; this scaling factor is typically used to scan the hierarchical structure of communities in a network.

There is more than one way to model the null model P_{uv}. Newman's approach is $P_{uv} = \frac{d_u d_v}{2m}$, which represents the random graph (no particular community structures) with the same empirical degree sequence. In theory, one can assume Poisson Distribution for node degree (like in the WSBM case). In the case of Newman's Q, this null model is built with respect to the empirical network degree. Furthermore, the tuning parameter is, by default, set at $\alpha = 1$, and the delta function is defined as

$$\delta(\sigma_u, \sigma_v) = \begin{cases} 1, & \text{if } \sigma_u = \sigma_v \\ 0, & \text{if } \sigma_u \neq \sigma_v \end{cases}$$

If *a priori* partition σ is known, then the modularity score can be written in a blockage format (only surviving terms in within communities: $\forall u, v \in V(G) \mid \sigma_u = \sigma_v = i \in [k]$) as follows:

$$\begin{aligned} Q(\sigma) &= \frac{1}{2m} \sum_{uv} \left\{ A_{uv} - \frac{d_u d_v}{2m} \right\} \delta(\sigma_u, \sigma_v) \\ &= \sum_{i=1}^{i=k} \left[\frac{\sum_{u,v \in i} A_{uv}}{2m} - \frac{\sum_{u,v \in i} d_u d_v}{(2m)^2} \right] \\ &= \sum_{i=1}^{i=k} \left[\frac{C_{ii}}{2m} - \sum_{u,v \in i} \frac{d_u}{2m} \frac{d_v}{2m} \right] \\ &= \sum_{i=1}^{k} \left[\frac{C_{ii}}{2m} - \left[\frac{s_i}{2m} \right]^2 \right] \\ &= \sum_{i=1}^{k} (\mathbb{P}_\sigma(ii) - \mathbb{P}^{Q}_{null}(ii)) \end{aligned}$$

Because:

$$\sum_{u,v \in i} A_{uv} = C_{ii}$$

and

$$\sum_{u,v\in i} d_u d_v = \sum_{\sigma_u=i} d_u^2 + 2\sum_{u\neq v} d_u d_v = (\sum_{\sigma_u=i} d_u)^2 = (s_1)^2$$

where s_1 is the total number of half-edges (stubs) that originate from nodes in community i.

We also look at the null model from another perspective: the event of a stub (half-edge) exists at node u with probability $\mathbb{P}_u(\text{stub}) = \frac{d_u}{2m}$, likewise, at node v with $\mathbb{P}_v(\text{stub}) = \frac{d_u}{2m}$. These two independent events need to happen sequentially to form an edge between node u and v with probability

$$\mathbb{P}^Q_{\text{null}} = \mathbb{P}_{uv}(\text{edge}) = \frac{d_u}{2m}\frac{d_v}{2m}, \forall \sigma_u = \sigma_v = i \in [k]$$

Note that here, no community indication is available for either node u or v, which implies a null model (i.e., random partition) of σ (ground-truth).

The Q-score can be applied to both binarized or weighted graphs. In this case, for each threshold value, the Q-score is computed for the weighted group-average FCs across Schaefer granularity levels. Maximizing modularity has been shown to unravel assortative communities, while SBM has been shown to uncover different types of communities, beyond assortative ones [5].

Philosophical Similarity between Log-likelihood Function and Q-score

In this section, we compare the NDC, DC WSBM, and Q-score approach by first revisiting their formulas:

(i) The NDC log-likelihood function (within communities):

$$L_{\text{within}} = \sum_i \frac{C_{ii}}{2m}\left\{\log\left[\frac{C_{ii}}{2m}\right] - \log\left[\frac{|\Omega_i|^2}{n^2}\right]\right\}$$

(ii) The DC log-likelihood function (within communities):

$$L_{\text{within}} = \sum_{i=1}^{k} \frac{C_{ii}}{2m}\left\{\log(C_{ii}/2m) - \log\left[\frac{s_i^2}{(2m)^2}\right]\right\}$$

(iii) Q-score modularity function, using community block format:

$$Q = \sum_{i=1}^{k}\left[\frac{C_{ii}}{2m} - \left[\frac{s_1}{2m}\right]^2\right]$$

It is very interesting (yet, not surprising) that the two most well-known methods for community detection are based on a similar principle of comparing a structureless counterpart (that has some similar topological characteristics) with the network at hand (which is hypothesized to have some latent structure of communities). In both

approaches, the hypothesized distribution of random variables Y' and Z' for within-community is actually the same:

$$\mathbb{P}^{\text{NDC-WSBM}} = \mathbb{P}^{\text{DC-WSBM}} = \mathbb{P}^{Q} = \frac{C_{ii}}{2m}$$

Obviously, there is a difference between the null model choice between the Q score and NDC WSBM approach as the latter does not feature the observed network degree sequence while Newman's Q method actually does. This shortcoming is resolved with the DC WSBM approach as mentioned in the previous section. In fact, the DC WSBM and Q score null model is actually the same. Specifically, for DC WSBM, the null model is

$$\mathbb{P}^{\text{DC-WSBM}}_{\text{null}} = \frac{s_i^2}{(2m)^2}$$

while for the modularity approach, it is also:

$$\mathbb{P}^{Q}_{\text{null}} = \frac{s_i^2}{(2m)^2}$$

What, then, is the shortcoming of the Q score approach? It does not emphasize what happens with the "between" community dynamics. To be clear, one can rewrite the Q score so that it reflects between-community edges as proposed by Fortunato [6] as follows:

$$\begin{aligned} Q &= \sum_{i=1}^{k} \left[\frac{C_{ii}}{2m} - \left[\frac{s_1}{2m} \right]^2 \right] \\ &= \frac{-1}{m} \left[\left\{ m - \frac{1}{2} \sum_i C_{ii} \right\} - \left\{ m - \sum_i \left(\frac{s_i^2}{4m} \right) \right\} \right] \\ &= \frac{-1}{m} \left[Cut - \mathbb{E}(Cut) \right] \end{aligned}$$

where $Cut = \left\{ m - \frac{1}{2} \sum_i C_{ii} \right\}$ being the number of inter-community edges and $\mathbb{E}(Cut)$ is its corresponding expected counterpart. Basically, modularity would like to maximize the within-community edges (equivalently, minimize the between-community edges). Hence, it biases towards assortative community assignments.

On the other hand, the log function of WSBM has also incorporated what happens between communities through $L_{between}$. This is why SBM inference method shines over traditional Q maximization techniques for its ability to uncover a more diverse range of community structures beyond assortative ones.

Statistics to compare Q_{max} and SBM-inference community detection approaches

Given the node set of N elements $S = \{s_i \mid i \in [N]\}$, to quantify the amount of shared information between two partitions (e.g., $\sigma^1 = \{\sigma^1_r \mid r \in [R]\}$ and $\sigma^2 = \{\sigma^2_c \mid c \in [C]\}$),

a common approach would be using mutual information score, which can be quantified as follows:

$$MI(\sigma^1, \sigma^2) = \sum_{r=1}^{R} \sum_{c=1}^{C} \mathbb{P}_{\sigma^1\sigma^2}(r, c) \log \frac{\mathbb{P}_{\sigma^1\sigma^2}(r, c)}{\mathbb{P}_{\sigma^1}(r)\mathbb{P}_{\sigma^2}(c)}$$

where R and C are the number of clusters in partition vectors σ^1 and σ^2, respectively; $\mathbb{P}_{\sigma^1\sigma^2}$ and $\mathbb{P}_{\sigma^1}$ are the joint and marginal probability distributions, respectively, between two discrete random variables representing two realized partitions. A typical initialization step is to build a contingency table which indicates the number of common nodes has in common between cluster σ_r^1 and σ_c^2:

$$\begin{pmatrix} n_{11} & n_{12} & \cdots & n_{1C} \\ \vdots & \cdots & \vdots & \cdots \\ n_{R1} & n_{R2} & \cdots & n_{RC} \end{pmatrix}$$

where n_{rc} represents the number of common entities between cluster σ_r^1 and σ_c^2; the row and column marginal sums are denoted as $\vec{a} = a_r$ and $\vec{b} = b_c$, respectively. By construction, $\sum_r a_r = \sum_c b_c = N$. The expected mutual information for a random partition with the same contingency table has a closed-form formula as proposed in [16]:

$$\mathbb{E}(MI(\sigma^1, \sigma^2)) = \sum_r \sum_c \sum_{\max(1, a_r+b_c-N)}^{\min(a_r, b_c)} \frac{n_{rc}}{N} \log \left\{ \frac{N \times n_{rc}}{a_r b_c} \right\} \\ \times \frac{a_r! b_c! (N - a_r)! (N - b_c)!}{N! n_{rc}! (a_r - n_{rc})! (b_c - n_{rc})! (N - a_r - b_c + n_{rc})!}$$

The entropy associated with the two partitions are:

$$H(\sigma^1) = \sum_{r=1}^{R} \mathbb{P}_{\sigma^1}(r) \log(\mathbb{P}_{\sigma^1}(r))$$

where $\mathbb{P}_{\sigma^1}(r) = \frac{|\sigma_r^1|}{N}$ is the probability that an element picked randomly from set S belongs to σ_r^1. Analogously,

$$H(\sigma^2) = \sum_{j=1}^{C} \mathbb{P}_{\sigma^2}(j) \log(\mathbb{P}_{\sigma^2}(j))$$

Finally, putting all components together, adjusted (for chance) normalized mutual information (AMI) can be computed as follows:

$$AMI = \frac{MI - \mathbb{E}(MI)}{\max(H(\sigma^1), H(\sigma^2)) - \mathbb{E}(MI)}$$

Note that there are other ways to average the independent entropy of the two partitions such as arithmetic. In this paper, we use the maximum between the two entropy quantities.

Graph Sequence and Required Topological Features for Recovery

A graph sequence is a mathematical series of graph ensembles generated by some fixed rules, denoted by $\{G_l\}$ where l is the sequence index. For example, one can generate an ER random graph sequence denoted as $G_t(t, p)$ with fixed p and its limiting graph denoted as $G_{t=\infty}(t, p)$, also known as a graphon. In recent developments in $SBM(k, p, W)$ theory, it is important to note the theoretical scaling characteristics of model parameters such as k, p, and W, and make necessary assumptions, i.e., which scales with t, and which stays constant as the graph size grows to ∞.

Firstly, it is common to assume that p and k do not scale with t; hence, the number of communities and their respective sizes do not grow with t [1]. In other words, communities are assumed to have linear sizes [1]. Moreover, matrix W has theoretical ties with an important topological characteristic of a graph sequence, such as the degree regime.

The importance of the degree regime lies in its relations with graph connectivity. There are two important degree regimes relevant for graph partition recoverability:

- **Constant Degree Regime**: In this regime, the connectivity pattern is fixed (independent of Schaefer granularity levels). Asymptotically, node degrees do not scale with graph size, i.e., $W = O(n^{-1})$. In random graph theory, this is the degree where an ER graph is expected to have a giant component. This regime satisfies the minimum requirement for the weak-recovery criteria, which will be formally defined later.
- **Diverging Degree Regime**: In this regime, the connectivity pattern varies with graph sequence sizes. Asymptotically, node degrees scale with graph size at a scalable factor s_t, i.e., $W = O(\log(n)n^{-1})$. In random graph theory, this degree regime generates a connected ER ensemble, in expectation. This regime satisfies the minimum requirement for exact recovery, which will be defined in a later section.

Data, Atlases, and Code Availability

Neuroimaging Data Acquisitions

The fMRI dataset used in this paper is available in the Human Connectome Project (HCP) repository (`http://www.humanconnectome.org/`), Released Q3. The processed functional connectomes obtained from this data and used for the current study are available from the corresponding author upon reasonable request. Please refer to the detailed descriptions below on the dataset and data processing.

We first describe the acquisitions of raw neuroimaging data from 409 Unrelated Subjects chosen from the list of 1200 participants by Essen et al. [14, 15] in the Human Connectome Project (HCP) release. This subset of participants ensures that no two participants have any family relations, sharing parents or being siblings. This selection is particularly critical to avoid any confounding effects in our subsequent analyses, such as group average analysis, due to family structures.

Per HCP protocol, all subjects gave written informed consent to the HCP consortium. The two resting-state functional MRI acquisitions (HCP filenames: rfMRI_REST$_1$ and rfMRI_REST$_2$ were acquired in separate sessions on two different days, with two distinct scanning patterns (left to right and right to left) in each day, [7], [15], and [14] for details. This release also includes data from seven different fMRI tasks: gambling (tfMRI_GAMBLING), relational reasoning (tfMRI_RELATIONAL), social (tfMRI_SOCIAL), working memory (tfMRI_WM), motor (tfMRI_MOTOR), language (tfMRI_LANGUAGE, including both a story-listening and arithmetic task), and emotion (tfMRI_EMOTION). Per [7], [4], three tasks MRIs are obtained: working memory, motor, and gambling. The local Institutional Review Board at Washington University in St. Louis approved all the protocols used during the data acquisition process. Please refer to [4, 7, 13] for further details on the HCP dataset.

Constructing Functional Connectomes

We used the standard HCP functional preprocessing pipeline, which includes artifact removal, motion correction, and registration to standard space, as described in [7, 13] for this dataset. For the resting-state fMRI data, we also added the following steps: global gray matter signal regression; a bandpass first-order Butterworth filter in both directions; z-scores of voxel time courses with outlier eliminations beyond three standard deviations from the first moment [9, 11].

For task fMRI data, the aforementioned steps are applied, with a relaxation for the bandpass filter [0.001 Hz, 0.25 Hz]. Starting from each pair of nodal time courses, Pearson correlation is used to fill out the functional connectomes for all subjects at rest and seven designated tasks. This would yield symmetrical connectivity matrices for all fMRI sessions.

Brain Atlases

The brain atlases used in this work are sequential, in the sense that their granularity increases, ranging from 100 nodes to 900 nodes (increment of 100 nodes each time), registered on the cortical surface of the brain. These sequential atlases are made possible thanks to the work of Schaefer and colleagues [12]. Similarly to references [2, 3], 14 sub-cortical regions were added, as provided by the HCP release (filename *Atlas_ROI2.nii.gz*). We accomplished this by converting this file from NIFTI to CIFTI format using the HCP Workbench software [(`http://www.humanconnectome.org/software/connectomeworkbench.html`), with the command `-cifti- create-label`. The resultant sizes of ROI-based connectomes are, hence, 114, 214,..., 914 nodes for rest and any given fMRI tasks. Mathematically, we denote the Schaefer parcellation sequence to be G_{t_ℓ} where $\ell \in [9]$ and $t_\ell = [114, 214, ..., 914]$.

Moreover, Schaefer parcellations are also coupled nicely with further subdivisions of Yeo's functional networks [17] so that the partition associated with a coarser Schaefer

graph is related to that of a finer-grained Schaefer one. For a fixed Schaefer granularity (indexed t_ℓ), we denote the corresponding Yeo's resting-state networks to be σ_{t_ℓ}.

For instance, let u_{114} be a node in the Schaefer graph with $n = 114$ nodes and a community label

$$\sigma_{114} = \{u_{114} \mapsto i \mid i \in [k], \forall u_{114} \in [114]\}.$$

Say, this is further subdivided into two nodes v'_{214} and v''_{214} in the next Schaefer graph in the sequence, i.e., G with $n = 214$ nodes, and that $u_{114} = \{v'_{214}, v''_{214}\}$. Then, it follows that:

$$\sigma_{214} = \{v'_{214} \mapsto i \quad \& \quad v''_{214} \mapsto i \mid i \in [k], \forall v_{214} \in [214]\}$$

In fact, we can generalize this as follows:

Definition 1. *Let l, q be graph sequence indices and σ be the network partition. If*

(i) $u_{n_l} = \cup u_{n_q} \quad s.t. \quad l < q, \quad u_{n_l} \in V(G_{n_l}), \quad u_{n_q} \in V(G_{n_q})$;

(ii) $\sigma_{n_l} = \{u_{n_l} \mapsto i \mid i \in [k]\}$

Then, $\sigma_{n_q} = \{u_{n_q} \mapsto i \mid i \in [k]\}$.

In practice, the subsequent divisions from coarser to finer granularity of Schaefer parcellations are not perfectly hierarchical in the sense that one node in the coarser parcellation does not perfectly parcellate into subsequently smaller ROIs in the finer one. Nonetheless, in this context, we can relax the condition (i) as follows: if the node associated with the coarser parcellation has the majority of spatial overlaps with the ones in subsequently finer parcellations of the Schaefer graph sequence, then they are assigned to the same resting state network.

Code Availability

The code to perform the *reconFC* procedure can be found at
https://github.com/ngcaonghi/fc_threshold_framework.

References

[1] E. Abbe. Community detection and stochastic block models: recent developments. *The Journal of Machine Learning Research*, 18(1):6446–6531, 2017.

[2] E. Amico and J. Goñi. Mapping hybrid functional-structural connectivity traits in the human connectome. *Network Neuroscience*, pages 1–17, 2018.

[3] E. Amico and J. Goñi. The quest for identifiability in human functional connectomes. *Scientific reports*, 8(1):8254, 2018.

[4] D. M. Barch, G. C. Burgess, M. P. Harms, S. E. Petersen, B. L. Schlaggar, M. Corbetta, M. F. Glasser, S. Curtiss, S. Dixit, C. Feldt, et al. Function in the human connectome: task-fmri and individual differences in behavior. *Neuroimage*, 80:169–189, 2013.

[5] R. F. Betzel, J. D. Medaglia, and D. S. Bassett. Diversity of meso-scale architecture in human and non-human connectomes. *Nature communications*, 9(1):1–14, 2018.

[6] S. Fortunato. Community detection in graphs. *Physics reports*, 486(3):75–174, 2010.

[7] M. F. Glasser, S. N. Sotiropoulos, J. A. Wilson, T. S. Coalson, B. Fischl, J. L. Andersson, J. Xu, S. Jbabdi, M. Webster, J. R. Polimeni, et al. The minimal preprocessing pipelines for the human connectome project. *Neuroimage*, 80:105–124, 2013.

[8] B. Karrer and M. E. Newman. Stochastic blockmodels and community structure in networks. *Physical review E*, 83(1):016107, 2011.

[9] D. Marcus, J. Harwell, T. Olsen, M. Hodge, M. Glasser, F. Prior, M. Jenkinson, T. Laumann, S. Curtiss, and D. Van Essen. Informatics and data mining tools and strategies for the human connectome project. *Frontiers in neuroinformatics*, 5:4, 2011.

[10] T. P. Peixoto. Nonparametric weighted stochastic block models. *Physical Review E*, 97(1):012306, 2018.

[11] J. D. Power, A. Mitra, T. O. Laumann, A. Z. Snyder, B. L. Schlaggar, and S. E. Petersen. Methods to detect, characterize, and remove motion artifact in resting state fmri. *Neuroimage*, 84:320–341, 2014.

[12] A. Schaefer, R. Kong, E. M. Gordon, T. O. Laumann, X.-N. Zuo, A. J. Holmes, S. B. Eickhoff, and B. T. Yeo. Local-global parcellation of the human cerebral cortex from intrinsic functional connectivity mri. *Cerebral cortex*, 28(9):3095–3114, 2018.

[13] S. M. Smith, C. F. Beckmann, J. Andersson, E. J. Auerbach, J. Bijsterbosch, G. Douaud, E. Duff, D. A. Feinberg, L. Griffanti, M. P. Harms, et al. Resting-state fmri in the human connectome project. *Neuroimage*, 80:144–168, 2013.

[14] D. C. Van Essen, S. M. Smith, D. M. Barch, T. E. Behrens, E. Yacoub, K. Ugurbil, W.-M. H. Consortium, et al. The wu-minn human connectome project: an overview. *Neuroimage*, 80:62–79, 2013.

[15] D. C. Van Essen, K. Ugurbil, E. Auerbach, D. Barch, T. Behrens, R. Bucholz, A. Chang, L. Chen, M. Corbetta, S. W. Curtiss, et al. The human connectome project: a data acquisition perspective. *Neuroimage*, 62(4):2222–2231, 2012.

[16] N. X. Vinh, J. Epps, and J. Bailey. Information theoretic measures for clusterings comparison: Variants, properties, normalization and correction for chance. *The Journal of Machine Learning Research*, 11:2837–2854, 2010.

[17] B. T. Yeo, F. M. Krienen, J. Sepulcre, M. R. Sabuncu, D. Lashkari, M. Hollinshead, J. L. Roffman, J. W. Smoller, L. Zöllei, J. R. Polimeni, et al. The organization of the human cerebral cortex estimated by intrinsic functional connectivity. *Journal of neurophysiology*, 106(3):1125–1165, 2011.
